# Integrative Analysis of Glycosylation-Related Genes Reveals Prognostic Subtypes, Immune Evasion, and Therapeutic Vulnerabilities in Lung Adenocarcinoma

**DOI:** 10.32604/or.2026.074013

**Published:** 2026-06-16

**Authors:** Yu-Wei Liu, Yung-Kuo Lee, Kai-Fu Chang, Chung-Bao Hsieh, Chih-Hsuan Chang, Ching-Chung Ko, Hui-Ru Lin, Chi-Jen Wu, Chien-Han Yuan, Sachin Kumar, Dahlak Daniel Solomon, Do Thi Minh Xuan, Neethu Palekkode, Ayman Fathima, Hung-Yun Lin, Chih-Yang Wang, Chih-Jen Yang, Yuen-Jung Wu

**Affiliations:** 1 Division of Thoracic Surgery, Department of Surgery, Kaohsiung Medical University Hospital, Kaohsiung Medical University, Kaohsiung, Taiwan; 2 Medical Laboratory, Medical Education and Research Center, Kaohsiung Armed Forces General Hospital, National Defense Medical University, Kaohsiung, Taiwan; 3 Division of Experimental Surgery Center, Department of Surgery, Tri-Service General Hospital, National Defense Medical University, Taipei City, Taiwan; 4 Institute of Medical Science and Technology, National Sun Yat-Sen University, Kaohsiung, Taiwan; 5 Department of General Surgery, Taipei City Hospital Zhongxing Branch, Taipei City, Taiwan; 6 Department of Medical Imaging, Chi-Mei Medical Center, Tainan, Taiwan; 7 Department of Health and Nutrition, Chia Nan University of Pharmacy and Science, Tainan, Taiwan; 8 School of Medicine, College of Medicine, National Sun Yat-Sen University, Kaohsiung, Taiwan; 9 Nursing Department, Kaohsiung Armed Forces General Hospital, National Defense Medical University, Kaohsiung, Taiwan; 10 College of Nursing, Kaohsiung Medical University, Kaohsiung, Taiwan; 11 Department of Otolaryngology, Kaohsiung Armed Forces General Hospital, National Defense Medical University, Kaohsiung, Taiwan; 12 Department of Otolaryngology, National Defense Medical University, Taipei City, Taiwan; 13 Graduate Institute of Cancer Biology and Drug Discovery, College of Medical Science and Technology, Taipei Medical University, Taipei City, Taiwan; 14 Ph.D. Program for Cancer Molecular Biology and Drug Discovery, College of Medical Science and Technology, Taipei Medical University, Taipei City, Taiwan; 15 Faculty of Applied Sciences and Biotechnology, Shoolini University of Biotechnology and Management Sciences, Bajhol, Himachal Pradesh, India; 16 Faculty of Pharmacy, Van Lang University, 69/68 Dang Thuy Tram Street, Ward 13, Binh Thanh District, Ho Chi Minh City, Vietnam; 17 Department of Biotechnology, Mother Teresa Women’s University, Kodaikanal, Tamil Nadu, India; 18 Computer Engineering with specialization in Artificial Intelligence and Machine Learning, Presidency University, Yelahanka, Bengaluru, Karnataka, India; 19 TMU Research Center of Cancer Translational Medicine, Taipei Medical University, Taipei City, Taiwan; 20 Traditional Herbal Medicine Research Center of Taipei Medical University Hospital, Taipei Medical University, Taipei City, Taiwan; 21 Pharmaceutical Research Institute, Albany College of Pharmacy and Health Sciences, Rensselaer, NY, USA; 22 Cancer Center, Wan Fang Hospital, Taipei Medical University, Taipei City, Taiwan; 23 Division of Pulmonary and Critical Care Medicine, Department of Internal Medicine, Kaohsiung Medical University Hospital, Kaohsiung Medical University, Kaohsiung, Taiwan; 24 School of Post-Baccalaureate Medicine, College of Medicine, Kaohsiung Medical University, No. 100, Tzyou 1st Road, Kaohsiung, Taiwan; 25 Department of Surgery, Kaohsiung Armed Forces General Hospital, National Defense Medical University, Kaohsiung, Taiwan

**Keywords:** Lung adenocarcinoma, glycosylation, prognostic biomarker, tumor microenvironment, immune evasion, single-cell RNA sequencing, immunohistochemistry, precision oncology

## Abstract

**Background:** Lung adenocarcinoma (LUAD) is the most common subtype of non-small cell lung cancer (NSCLC) and remains a leading cause of cancer-related mortality worldwide. Aberrant glycosylation contributes to tumor progression by regulating receptor signalling, immune evasion, and metastatic. However, the prognostic and therapeutic relevance of glycosylation-related genes (GRGs) in LUAD has not been comprehensively defined. Therefore, this study aimed to comprehensively evaluate GRG-associated molecular subtypes and their clinical and therapeutic relevance in LUAD. **Methods**: GRGs were curated from multiple public databases and integrated with transcriptomic and clinical data from The Cancer Genome Atlas LUAD cohort (TCGA-LUAD) with validation in Gene Expression Omnibus (GEO) datasets. Consensus clustering, pathway enrichment, and immune profiling were used to identify glycosylation-associated subtypes. A glycosylation activity scoring (Glyco. marker) was developed to quantify glycosylation features. Drug response prediction was analyzed using OncoPredict and the Genomics of Drug Sensitivity in Cancer (GDSC) database. Single-cell RNA sequencing (scRNA-seq) was analyzed to evaluate cell-type-specific GRG expression. Selected proteins were by immunohistochemistry (IHC) in LUAD tissue microarrays. **Results:** GRG expression stratified 513 LUAD patients into four molecular clusters with distinct clinical and immune characteristics. The Glyco.High group showed elevated expression of MGAT5 (mannosyl (α-1,6)-glycoprotein β-1,6-N-acetylglucosaminyltransferase), ST6GAL1 (β-galactoside α-2,6-sialyltransferase 1), GALNT7 (polypeptide N-acetylgalactosaminyltransferase 7), and FUT8 (fucosyltransferase 8), frequent tumor protein p53 (TP53) mutations, increased immune checkpoint expression, and enrichment of regulatory T cells. The Glyco. marker score predicted overall survival and was associated with stemness signatures. Drug response prediction suggested reduced sensitivity to platinum chemotherapy and epidermal growth factor receptor (EGFR) inhibitors but increased sensitivity to phosphoinositide 3-kinase/protein kinase B/mechanistic target of rapamycin (PI3K/AKT/mTOR) inhibitors. **Conclusion:** GRG-based molecular stratification identifies clinically distinct LUAD subtypes associated with immune regulation, tumor stemness, and therapeutic response. The Glyco. marker system provides a potential framework for prognostic assessment and precision oncology strategies in LUAD.

## 1 Introduction

Lung adenocarcinoma (LUAD), the most common subtype of non-small cell lung cancer (NSCLC), accounts for nearly 40% of lung cancer cases and remains a leading cause of cancer-related morbidity and mortality worldwide [1,2,3]. Despite significant advances in surgery, chemotherapy, targeted therapy, and immunotherapy, the 5-year survival rate of LUAD patients remains dismal, largely due to late diagnosis, acquired drug resistance, and pronounced tumor heterogeneity. Comprehensive molecular stratification is therefore essential for improving patient prognosis and guiding precision medicine [4,5,6].

Among the diverse biological processes contributing to LUAD progression, glycosylation, a post-translational modification involving the attachment of glycans to proteins and lipids, has emerged as a crucial regulator of cancer biology [7,8]. Aberrant glycosylation is widely recognized as a hallmark of malignancy, influencing protein folding, receptor stability, intracellular signaling, immune evasion, and metastasis. Specific alterations, such as increased β-1,6-N-acetylglucosamine branching mediated by mannosyl (α-1,6-glycoprotein) β-1,6-N-acetylglucosaminyltransferase (MGAT5), hypersialylation via β-galactoside α-2,6-sialyltransferase (ST6GAL1), and O-glycan elongation through polypeptide N-acetylgalactosaminyltransferase (GALNT) family members, have been associated with tumor aggressiveness and poor clinical outcomes [9,10]. Importantly, glycosylation modifications also regulate immune checkpoint function; for instance, N-linked glycosylation stabilizes programmed death-ligand 1 (PD-L1) on the tumor surface, enhancing its ability to suppress T cell–mediated cytotoxicity. This indicates that glycosylation not only drives intrinsic tumor growth but also shapes the tumor immune microenvironment (TME) [11,12,13].

Emerging studies have emphasized the interplay between glycosylation and LUAD therapeutic resistance. Epidermal growth factor receptor (EGFR), anaplastic lymphoma kinase (ALK), and other receptor tyrosine kinases (RTKs) undergo extensive glycosylation, which modulates ligand binding, dimerization, and downstream signaling [14]. Glycan remodeling has been shown to contribute to resistance against tyrosine kinase inhibitors (TKIs) and platinum-based chemotherapy by enhancing receptor recycling, DNA repair pathways, and epithelial-mesenchymal transition (EMT) [15,16]. Moreover, aberrant glycosylation patterns generate tumor-associated carbohydrate antigens (TACAs), such as sialyl-Tn and Lewis antigens, which are linked to metastatic dissemination and immune escape. These findings suggest that glycosylation is not merely a bystander process but a driver of LUAD progression and treatment outcomes [17,18,19].

Despite these insights, a systematic understanding of glycosylation-related genes (GRGs) and their prognostic implications in LUAD remains incomplete [20,21]. Most existing studies have focused on individual enzymes or pathways, without integrating multi-database evidence to comprehensively delineate glycosylation-associated subtypes [22,23,24,25,26]. Furthermore, the relationship between GRGs, mutational landscapes, immune infiltration, stemness phenotypes, and therapeutic response has not been fully elucidated. Given the heterogeneity of LUAD and the multifaceted role of glycosylation in tumor biology, there is an urgent need to explore glycosylation signatures as both prognostic biomarkers and therapeutic targets [27,28,29]. In this context, the present study examines glycosylation-related gene expressions as an indirect representation of glycosylation-associated transcriptional programs, which enables systematic investigation of glycosylation-linked molecular patterns at the cohort level. This approach is intended to characterize broad glycosylation-related pathway activity rather than to directly measure glycan structures or post-translational glycosylation states.

In this study, we systematically curated GRGs from four authoritative resources—Gene Set Enrichment Analysis Molecular Signatures Database (GSEA-MSigDB), Enrichr, Harmonizome 3.0, and GlycoGene database to perform integrative analyses in LUAD. By applying consensus clustering, survival modeling, immune profiling, and drug sensitivity prediction, we aimed to identify robust glycosylation-defined LUAD subtypes with distinct molecular and clinical characteristics. Additionally, we constructed a glycosylation scoring system to quantify glycosylation activity at both bulk and single-cell resolution, explored its association with stemness and immune evasion, and employed a signature-related gene analysis (SRGA) framework to discover novel GRG markers (Supplementary Fig. S1). Collectively, our work seeks to provide a comprehensive understanding of the role of glycosylation-linked molecular heterogeneity in LUAD, offering insights that may support biomarker development and precision oncology strategies.

## 2 Methods

### 2.1 Collection of Glycosylation-Related Genes (GRGs)

Human glycosylation-related genes (GRGs) were systematically curated from four well-established and publicly available resources: the Gene Set Enrichment Analysis Molecular Signatures Database (GSEA-MSigDB), Enrichr, Harmonizome 3.0, and the GlycoGene Database. These resources were selected because they integrate curated pathway annotations, experimentally supported gene sets, literature-based associations, and expert-annotated glycosylation enzymes and regulators, thereby capturing complementary aspects of glycosylation biology. Gene sets were retrieved using glycosylation-related keywords including glycosylation, glycan biosynthesis, glycan modification, glycoprotein and glycosyltransferase processing was retrieved. Data was accessed in October 2025.

Gene symbols from all databases were standardized according to official HGNC nomenclature to ensure cross-database consistency. All sources were initially treated equally during integration. Duplicate entries were removed, and genes appearing in multiple databases were prioritized as higher-confidence candidates to reduce the likelihood of weakly supported associations. After integration and de-duplication, a total of 421 non-redundant GRGs were retained for downstream analyses. The final GRG set comprised multiple functional categories, including glycosyltransferases, glycosidases, glycan-processing enzymes, glycoprotein biosynthesis regulators, and proteins involved in glycan transport and trafficking. These genes were used for molecular clustering, pathway enrichment analysis, glycosylation scoring, and single-cell validation. The curated GRG set was intended to capture xglycosylation-associated transcriptional programs and pathway-level activity, rather than directly measure glycan structures or post-translational glycosylation states.

### 2.2 Data Acquisition and Preprocessing

Transcriptome expression profiles and corresponding clinical information of LUAD (LUAD) patients were obtained from The Cancer Genome Atlas (TCGA-LUAD) through the UCSC Xena browser (accessed in October 2025). For external validation, independent LUAD cohorts were downloaded from the Gene Expression Omnibus (GEO) database, including GSE31210. TCGA RNA-seq expression data were obtained as HTSeq count matrices, which were converted to transcripts per kilobase million (TPM) and log2 transformed for downstream analyses. GEO microarray data were processed and analyzed sing the normalized expression matrices provided by the original studies. Because TCGA RNA-seq data, and GEO microarray datasets originate from fundamentally different sequencing platforms, the datasets were processed and analyzed separately using platform-specific normalization procedures and were not directly merged at the expression level. Therefore, cross-platform batch correction methods (such as sva or ComBat) were not applied. Within-cohort batch effects were evaluated using principal component analysis (PCA) to confirm that no dominant technical batch effects were present.

Somatic mutation data including single nucleotide variations (SNVs) and small insertions/deletions (INDELs), as well as copy number variation (CNV) profiles for LUAD were retrieved from the Genomic Data Commons (GDC) portal. Samples were excluded if they lacked key clinical annotations (such as survival status or follow-up time) or exhibited incomplete sequencing information [30].

### 2.3 UALCAN-Based Expression Analysis

The UALCAN web portal (http://ualcan.path.uab.edu) was used to evaluate the mRNA expression levels of selected GRGs (MGAT5, GALNT7, FUT8, ATP11B, and ST6GAL1) in LUAD. The analysis was performed using the TCGA-LUAD RNA-sequencing dataset available through the UALCAN platform (accessed in November 2025). Expression values normalized as normalized as transcripts per million (TPM), were used to compare gene expression between normal lung tissues and tumor samples.

Gene expression levels were further stratified according to tumor stage, patient race, gender, TP53 mutation status, smoking history, and histological subtypes. All analyses were conducted using the default UALCAN parameters, implemented in the UALCAN platform, and results were visualized as boxplots [31]. Statistical testing and visualization were performed using the algorithms integrated within the UALCAN portal and no additional multiple testing correction was applied beyond the default procedures provided by UALCAN.

### 2.4 Consensus Molecular Clustering of LUAD Patients

To classify LUAD patients based on GRG expression profiles, unsupervised consensus clustering was performed using the ConsensusClusterPlus R package (version 1.66.0). The analysis was conducted using the TCGA-LUAD cohort, and only tumor samples were included for clustering. The clustering algorithm was set to partitioning around medoids (PAM) with Pearson correlation distance, using 80% sample resampling (pItem = 0.8) and 1000 iterations to ensure clustering stability.

The optimal number of clustering number (k) was determined by evaluating the cumulative distribution function (CDF) curves and corresponding delta area curves. which measures the relative increase in cluster stability across different k values. Clustering robustness was further evaluated using principal component analysis (PCA) and silhouette width. PCA was used to visualize the separation of clusters in a reduced dimensional space, whereas silhouette width provided a quantitative measure of cluster cohesion and separation, thereby enabling complementary evaluation of clustering stability [32].

### 2.5 Gene Set Enrichment and Pathway Analysis

Gene set variation analysis (GSVA) and single-sample gene set enrichment analysis (ssGSEA) were applied to estimate the activity of glycosylation-related pathways and immune-associated signatures. Analyses were performed using the GSVA R package (version 1.50.0), with the Gaussian kernel function applied for RNA sequencing data and expression values normalized using log2-transformed transcripts per kilobase million (TPM) matrices.

Glycosylation-related gene sets were obtained from MSigDB, whereas immune and stromal gene signatures were curated from Tumor and Immune System Interaction Database (TISIDB) and the Immuno-Oncology Biological Research (IOBR) databases. To minimize redundancy and potential bias, curated gene signatures were subjected to preprocessing steps including removal of duplicated gene sets, standardization of gene symbols according to HGNC nomenclature, and exclusion of gene sets with very small gene counts. Differential pathway activities between subgroups were analyzed using the clusterProfiler R package (version 4.10.0), with false discovery rate (FDR) < 0.05 considered significant [33,34].

### 2.6 Somatic Mutation and Copy Number Variation Analysis

The maftools R package (version 2.18.0) was used to visualize mutational landscapes, calculate tumor mutation burden (TMB), and mutant-allele tumor heterogeneity (MATH). Significant co-occurrence and mutually exclusive mutations across GRG-defined subgroups were evaluated by the chi-square test. Copy number variation (CNV) analysis was performed using the Genomic Identification of Significant Targets in Cancer (GISTIC2) algorithm to identify significantly amplified or deleted chromosomal regions. The analysis was conducted using standard GISTIC2 parameters, including amplification and deletion thresholds of ±0.1, a confidence level of 0.95, and a false discovery rate (FDR) threshold of 0.25 to define significant genomic events. Differences in mutation frequencies between clusters were assessed using chi-square or Fisher’s exact tests, with *p* < 0.05 considered statistically significant.

### 2.7 Tumor Microenvironment and Immune Infiltration Analysis

The immune and stromal components of the tumor microenvironment (TME) were evaluated using the Estimation of STromal and Immune cells in MAlignant Tumor tissues using Expression data (ESTIMATE) algorithm implemented in the estimate R package (version 1.0.13), which calculates Immune Score, Stromal Score, and ESTIMATE Score. Relative abundances of infiltrating immune cells were quantified by single-sample gene set enrichment analysis (ssGSEA) implemented in the Gene Set Variation Analysis R package (GSVA, version 1.50.0) based on immune-related gene signatures. Expression levels of immune checkpoint molecules, including programmed cell death protein 1 (PD-1), programmed death-ligand 1 (PD-L1), cytotoxic T-lymphocyte-associated protein 4 (CTLA-4), lymphocyte activation gene 3 (LAG3), and T cell immunoreceptor with Ig and ITIM domains (TIGIT) was compared among subgroups using the Wilcoxon rank-sum test as appropriate. To account for multiple comparisons, false discovery rate (FDR) correction was applied using the Benjamini-Hochberg method.

In addition, pathway signatures related to immune exhaustion, immune suppression, and immune exclusion were evaluated to assess potential immunotherapy responsiveness [35].

### 2.8 Construction of Glycosylation Scoring System

To construct a robust glycosylation scoring system, glycosylation-related genes (GRGs) shared between the TCGA-LUAD cohort, and the independent GEO cohort (GSE31210) were first identified. A total of 496 overlapping GRGs were retained for downstream analysis. Fuzzy c-means clustering was performed using the *Mfuzz* R package (version 2.60.0) to identify stable expression patterns associated with glycosylation features across patients. Expression data were standardized prior to clustering. The optimal number of clusters was determined empirically based on cluster separation and stability. The fuzzification parameter (*m*) was set to 1.25, and genes with a cluster minimum cluster membership value ≥ 0.5, were considered to show a strong association with a given cluster [36]. 

Genes exhibiting both high cluster membership and substantial expression variability across samples (assessed by standard deviation of normalized expression values) were selected for signature construction. Based on these criteria, 100 genes were retained to construct the Glyco.marker signature (complete gene list provided in Supplementary Table S1). Alternative signature sizes were explored during preliminary analyses; however, the 100-gene signature provided stable clustering performance and reproducible glycosylation scoring across datasets. The Glyco. marker score for each sample was calculated as the as the mean normalized expression value of the genes included in the Glyco.marker signature. Patients were subsequently stratified into Glyco. High and Glyco.Low groups according to the median score within each cohort.

### 2.9 Stemness Analysis

Stemness features were quantified using enrichment scores derived from established embryonic stem cell-like signatures including BHATTACHARYA_ESC, and WONG_ESC_CORE, via calculated using ssGSEA implemented in the GSVA R package (version 1.50.0). Expression matrices were log2-transformed transcripts per kilobase million (TPM) values, and enrichment scores were computed using the default ssGSEA parameters within the GSVA framework. Correlation heatmaps were generated to illustrate associations between GRG expression and stemness indices. Correlation analyses were performed using Spearman’s rank correlation coefficient, and multiple testing correction was applied using the false discovery rate (FDR) method. Comparisons of stemness enrichment across GRG-based subgroups and Glyco.High and Glyco.Low groups were performed using the Wilcoxon rank-sum tests as enrichment scores often deviate from the normal distribution. Effect sizes were also considered to support the interpretation of group differences. All statistical analyses were conducted in R (version 4.4.1) [37].

### 2.10 Drug Sensitivity Prediction

Drug response predictions were performed using the OncoPredict R package (version 0.2.0), which estimates drug sensitivity based on transcriptomic similarity to cell lines from the Genomics of Drug Sensitivity in Cancer (GDSC) database. Gene expression matrices were log2-transformed transcripts per kilobase million (TPM) values prior to prediction. Predicted half-maximal inhibitory concentration (IC50) values were estimated for each patient sample using the default parameters implemented in the OncoPredict framework. 

To explore the association between glycosylation status and predicted drug response, Pearson correlation analysis was applied to evaluate the relationship between Glyco. marker scores and estimated IC50 values across candidate therapeutic agents. Associations with an absolute correlation coefficient (|r| > 0.4) and a two-sided *p* value, *p* < 0.05 were considered indicative of moderate-to-strong relationships to control for multiple comparisons across multiple drugs, false discovery rate (FDR) correction using the Benjamini-Hochberg method was applied [38,39,40]. These analyses were conducted in an exploratory, hypothesis-generating framework. Predicted drug sensitivity values represent computational estimates derived from GDSC-trained pharmacogenomic models rather than experimentally measured responses and therefore do not constitute direct clinical or pharmacological validation.

### 2.11 Identification of Novel GRG Markers Using SRGA

Signature-related gene analysis (SRGA) was performed as an integrative prioritization framework to identify candidate regulators associated with glycosylation-driven phenotypes. Gene-level associations with the Glyco.marker scores were first evaluated using Spearman’s rank correlation analysis which is suitable for transcriptomic data because it does not assume a normal distribution and is robust to outliers. Genes showing significant association with Glyco.marker scores were further examined for enrichment across hallmark oncogenic pathways using pathway signature scores. Candidate genes were then evaluated for prognostic relevance using univariate Cox proportional hazards regression and Kaplan-Meier survival analysis. To prioritize candidate regulators, genes were ranked based on integrated evidence including correlation strength with Glyco. marker scores, connectivity with enriched oncogenic pathways, and prognostic significance in survival analyses. Genes demonstrating consistent association across these analytical layers were considered potential glycosylation-associated regulators. It should be noted that SRGA represents an integrative prioritization approach and does not imply direct causal relationships [41].

### 2.12 Single-Cell Data Acquisition and Processing

LUAD single-cell RNA-sequencing (scRNA-seq) data in .h5 format together with cell type annotation results were obtained from the Tumor Immune Single-Cell Hub (TISCH) database (http://tisch.comp-genomics.org/) [42]. All analyses were conducted in R (v4.4.1) using MAESTRO (version 1.6.0) and Seurat packages (version 5.0.1).

Quality control filtering was performed to remove low-quality cells. Cells with fewer than 200 detected genes, more than 6000 detected genes, or mitochondrial gene expression exceeding 10% were excluded. Genes expressed in fewer than three cells were also removed. Data normalization and scaling were conducted using the standard Seurat workflows, including NormalizeData and ScaleData. Where multiple samples were present, batch effects were mitigated using Seurat-anchor based integration pipeline, implemented through the FindIntegrationAnchors and IntegrateData functions to align shared biological signals while minimizing technical variability. Dimensionality reduction was performed using principal component analysis (PCA), followed by t-distributed stochastic neighbor embedding (t-SNE) for visualization of cellular heterogeneity. Cell identities were initially assigned according to original TISCH annotations and canonical lineage marker genes were used to validate and refine these cell type assignments when necessary.

The single-cell analysis was designed to provide cell type-specific contextual validation of glycosylation-related gene expression rather than to perform patient-level stratification. Because the scRNA-seq datasets were not matched to the bulk transcriptomic profile, Glyco.High and Glyco.Low classifications could not be directly inferred at the single-cell level. Representative GRGs including ST6GAL1, MGAT5, GALNT7, FUT8, and ATP11B were selected based on their relevance to glycosylation pathways and their differential expression patterns observed in bulk transcriptomic analyses. Their expression distributions across annotated cell populations were visualized using t-SNE feature plots and summarized using mean expression bar charts.

### 2.13 Protein-Level Validation

Protein expression of GRGs was evaluated using LUAD tissue microarrays (TMAs) data obtained from the Human Protein Atlas (HPA) database (https://www.proteinatlas.org). Representative IHC images for ST6GAL1, MGAT5, GALNT7, FUT8, and ATP11B in LUAD and corresponding normal lung tissues were retrieved from the HPA resource to provide protein-level validation of transcriptomic findings. The Human Protein Atlas generates IHC data using standardized staining protocols, validated antibodies, and quality-controlled experimental pipelines. Staining intensity was interpreted according to the Human Protein Atlas annotation system, which classifies protein expression into four categories: High, Medium, Low, and Not detected, based on staining intensity and the proportion of positively stained cells within tissue sections. These annotations were used to summarize GRG protein expression patterns in LUAD tissues compared with normal lung tissues [43].

### 2.14 Statistical Analysis

All statistical analyses were performed using R software (version 4.4.1). For comparisons between two groups, the Wilcoxon rank-sum test was applied, while comparisons among more than two groups were conducted using one-way analysis of variance (ANOVA. Survival outcomes were evaluated using the Kaplan-Meier method and with differences assessed by the log-rank test. Multivariate Cox proportional hazards regression was employed to identify independent prognostic factors. Somatic mutation frequencies across glycosylation-defined clusters were compared using chi-square tests or Fisher’s exact tests, as appropriate based on expected cell counts. These analyses were conducted to identify cluster-enriched mutational patterns in an exploratory framework. Where applicable, multiple testing correction was applied using the false discovery rate (FDR) method. Correlation analyses were performed using Spearman’s rank correlation coefficient unless otherwise specified. All statistical tests were two-sided, with *p* < 0.05 or FDR < 0.05 was considered statistically significant. For analysis performed using publicly available web-based platforms (including UALCAN and drug sensitivity prediction tools), statistical testing and visualization were conducted using the default algorithms and parameters implemented by the respective platforms, as described in their original publications.

Data visualization was performed using the survival (version 3.6–4), survminer (version 0.4.9), and ggplot2 (version 3.5.1) R packages. Conceptual schematics were created using BioRender under a licensed academic account.

## 3 Results

### 3.1 Transcriptome Profiles of GRGs Defined Four Robust LUAD Clusters

**Figure 1:**
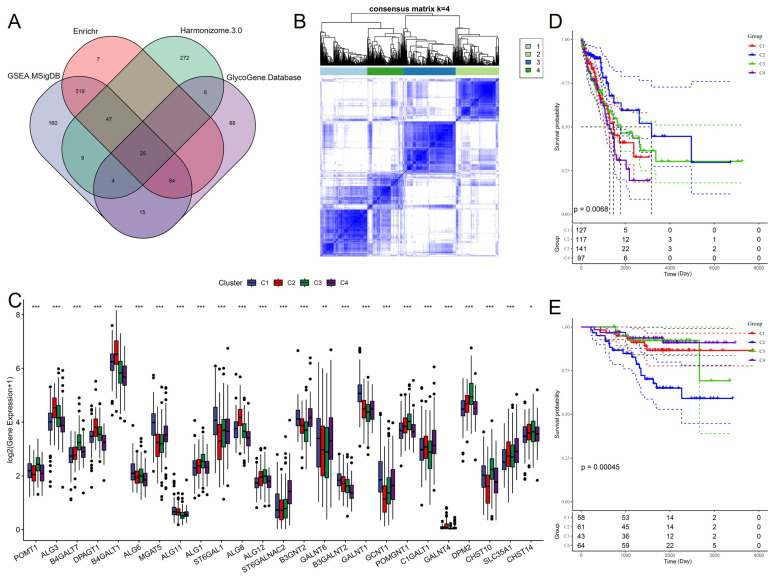
**Identification and characteristics of four lung adenocarcinoma (LUAD) clusters based on glycosylation-related genes (GRGs).** (**A**) Venn diagram showing the overlap of GRGs collected from four independent databases. (**B**) Consensus clustering matrix for k = 4 showing stable separation of LUAD samples into four molecular clusters (C1–C4). (**C**) Differential expression patterns of representative GRGs among the four clusters. (**D**) Kaplan–Meier survival analysis showing overall survival (OS) of the GRG-defined clusters in the TCGA-LUAD cohort. (**E**) Kaplan–Meier overall survival (OS) of the GRG-defined clusters in the independent Gene Expression Omnibus (GEO) cohort, confirming prognostic stratification. *p < 0.05, **p < 0.01, ***p < 0.001.

We first comprehensively curated glycosylation-related genes (GRGs) from four established resources (GSEA-MSigDB, Enrichr, Harmonizome 3.0, and GlycoGene Database) and retained 421 non-redundant candidates after removing duplicate entries. The Venn diagram demonstrated both shared and unique contributions of each database, with 25 genes consistently identified across all sources (Fig. 1A), suggesting their fundamental involvement in glycosylation-associated biological processes. Using unsupervised consensus clustering based on GRG expression profiles, LUAD patients were stratified into four distinct clusters (C1–C4). The consensus matrix and cumulative distribution function curves supported the robustness and stability of the four-cluster solution (Fig. 1B). Comparative analysis of clinical characteristics revealed that the distribution of pathological stage and nodal status differed significantly among the four clusters (chi-square test, *p* < 0.05), indicating an association between glycosylation-associated molecular subtypes and clinical disease features. Importantly, the distribution of patient clinical features, including pathological stage and nodal status, showed significant differences across clusters (chi-square test, *p* < 0.05), indicating an association between glycosylation-associated molecular subtypes and clinical disease characteristics. Differential expression analysis revealed that several cluster-specific patterns of glycosylation-related genes, including glycosyltransferases (MGAT5, ST6GAL1, FUT8), sialyltransferases (ST3GAL family, ST6GALNAC2), and O-glycan initiators (GALNT1, GALNT7) displayed cluster-specific patterns (Fig. 1C). Cluster C3 and C4 were characterized by elevated expression of genes involved in N-glycans branching and terminal sialylation transcriptional features previously associated with epithelial–mesenchymal transition (EMT), metastatic potential, and immune modulation. In contrast, C1 displayed a relatively balanced expression of glycosylation-related gene, with enrichment of enzymes involved in early glycan assembly and protein quality control, such as ALG family members, suggesting a less pronounced glycosylation-associated phenotype. Survival analysis demonstrated the prognostic value of glycosylation-associated clustering. In the TCGA-LUAD cohort, Cluster C1 was associated with the poorest overall survival whereas Cluster C2 showed the most favorable outcome (Fig. 1D), whereas in the independent GEO cohort, Cluster C2 showed the least favorable survival outcome (Fig. 1E). Notably, Cluster C1 displayed an intermediate survival pattern in both cohorts rather than consistently representing a favorable-risk group. Although the specific high-risk cluster differed between cohort, glycosylation-associated subtypes consistently stratified patients by overall survival across independent datasets, indicating reproducible prognostic separation rather than dependence on a single cluster label. On the other hand, Cluster C1 was consistently associated with a more favorable prognosis. These observations are in line with prior studies reporting that transcriptional programs linked to complex N-glycan branching and terminal sialylation are associated with aggressive tumor behavior and immune modulation, including stabilization of immune checkpoint molecules such as PD-L1 [44,45]. Collectively, these results indicate that GRG expression profiles define molecularly distinct LUAD subtypes with prognostic significance and support their potential utility for patient stratification. The identification of glycosylation-associated LUAD clusters provides a framework for future investigation of glyco-immune interactions and the development of glycosylation-informed therapeutic strategies in LUAD.

To further validate the clinical relevance of representative GRGs, we examined the expression patterns of MGAT5, GALNT7, FUT8, ATP11B, and ST6GAL1 across multiple clinicopathological subgroups of LUAD using TCGA data accessed via the UALCAN platform. Gene expression was compared between normal lung tissues and LUAD samples and further stratified by tumor stage, patient race, gender, TP53 mutation status, smoking history, and histological subtypes. As shown in Supplementary Figs. S2–S6, all five genes exhibited higher expression in LUAD compared with normal lung tissues, with varying degrees of heterogeneity across clinical subgroups. Notably, MGAT5, GALNT7, and FUT8 display stage-associated and histology-dependent expression patterns, consistent with their roles in glycosylation remodeling during tumor progression. ATP11B and ST6GAL1 also showed subgroup-specific variability, supporting their potential involvement in LUAD molecular heterogeneity. These findings further substantiate the clinical relevance of the glycosylation-associated genes identified in our integrative analyses.

### 3.2 Mutational Characteristics of Glycosylation-Related LUAD Clusters

**Figure 2:**
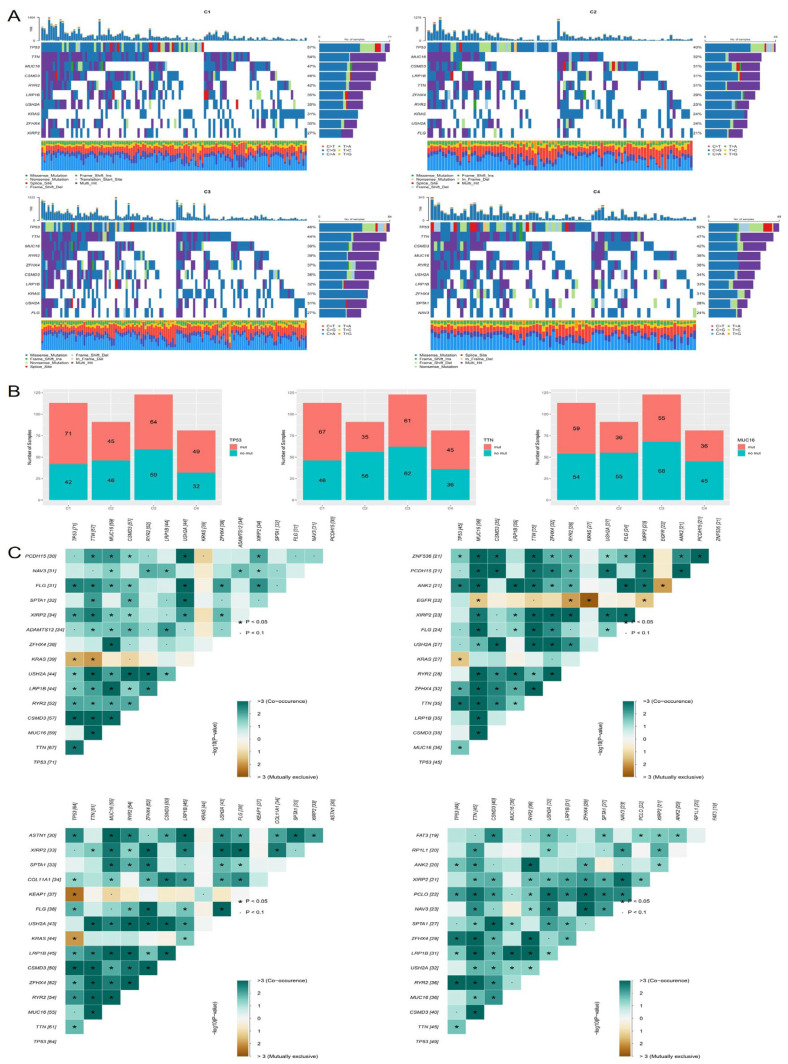
**Mutational landscape across four LUAD clusters defined by glycosylation-related genes (GRGs).** (**A**) Waterfall plots showing single nucleotide variations (SNVs) and small insertions/deletions (INDELs) in the four clusters. (**B**) Mutation frequencies of key LUAD driver genes, including tumor protein p53 (TP53), titin (TTN), and mucin 16 (MUC16), across the four clusters. (**C**) Co-occurrence and mutual exclusivity of somatic mutations among the top mutated genes in LUAD, with significant associations indicated. *p < 0.05, *p* < 0.1.

To characterize the genomic features underlying glycosylation-defined LUAD subtypes, we compared somatic mutation profiles across the four GRG-based clusters. Across all clusters, TP53, TTN, and MUC16, were the most frequently mutated genes, consistent with known mutational patterns in LUAD. While these genes were commonly altered across clusters, their mutation frequencies showed moderate variation, reflecting subtype-associated mutational heterogeneity rather than mutually exclusive genomic drivers (Fig. 2A,B). TP53 mutations were prevalent in all clusters, with comparable mutation frequencies observed in Clusters C1 and C4, indicating that TP53 alteration represents a shared genomic feature rather than a distinguishing event between these subtypes. Similarly, TTN and MUC16 mutations were most frequently observed in Cluster C1, with lower and more variable frequencies across the remaining clusters. These highly mutated genes likely reflect background mutational burden and tumor immunogenicity rather than cluster-specific oncogenic dependence.TP53 mutation frequencies were high across all clusters, with only minor differences between C1 and C4 that are unlikely to be biologically or statistically meaningful. This enrichment is concordant with the poor clinical outcome associated with C4 and suggests a link between disrupted genomic surveillance and aggressive glycosylation phenotypes. In contrast, TTN and MUC16 mutations were most frequently observed in Cluster C1, with lower frequencies in the remaining clusters, consistent with their large gene size and high background mutation rates. Additional LUAD-relevant genes, including KEAP1, KRAS, and CSMD3, also displayed heterogeneous mutation patterns across clusters, further underscoring molecular diversity. Notably, several glycosylation-related regulators are located within chromosomal regions that are frequently altered in LUAD, such as FUT family members at 19q13 and GALNT genes at 12q13. The co-occurrence of these regions with canonical oncogenic alterations suggests that chromosomal instability may contribute to coordinated deregulation of glycosylation pathways. Co-mutation and mutual exclusivity analyses provided additional insight into cluster-specific genomic interactions (Fig. 2C). Significant co-occurrence was observed between TP53 and CSMD3 in C4, KRAS and STK11 in C2, and KEAP1 and NFE2L2 in C3, suggesting cooperative oncogenic programs within distinct glycosylation contexts. In contrast, classical mutually exclusive relationships, such as EGFR versus KRAS mutations, were preserved across all clusters, highlighting their fundamental and glycosylation-independent roles in LUAD biology. Importantly, several of the enriched mutation patterns may indirectly influence glycosylation-associated transcriptional programs through pathway modulation. For example, alterations in the KEAP1/NFE2L2 axis are known to regulate oxidative stress responses and metabolic reprogramming, which can affect flux through the hexosamine biosynthetic pathway and downstream glycan synthesis. Likewise, mutations affecting EGFR signaling may interact with receptor glycosylation status to modulate receptor stability and downstream signaling intensity. Collectively, these results demonstrate that GRG-defined LUAD clusters are associated not only with distinct transcriptomic programs but also with characteristic mutational landscapes. While these associations are correlative, they highlight potential points of crosstalk between oncogenic signaling pathways genomic instability, and glycosylation-associated transcriptional programs that warrant further functional investigation.

### 3.3 Immune Profiling of LUAD Clusters Defined by GRGs

**Figure 3:**
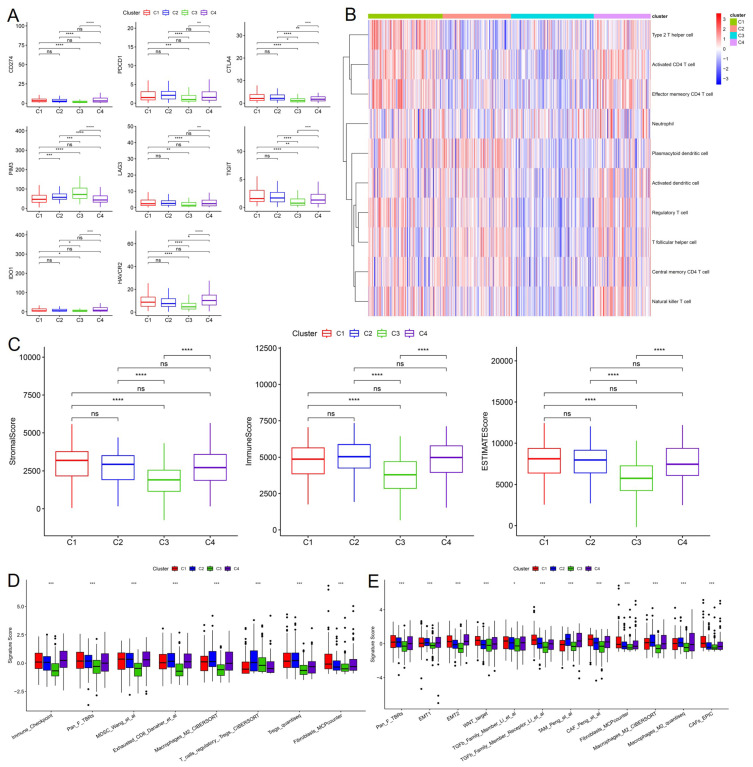
**Tumor immune microenvironment of glycosylation-related gene (GRG)-defined LUAD clusters.** (**A**) Expression of eight immune checkpoint molecules, including programmed death-ligand 1 (PD-L1), programmed cell death protein 1 (PD-1), cytotoxic T-lymphocyte–associated protein 4 (CTLA4), lymphocyte-activation gene 3 (LAG3) across the four LUAD clusters. (**B**) Heatmap showing relative infiltration of immune cell subsets estimated by ingle-sample gene set enrichment analysis (ssGSEA). (**C**) Distribution of stromal score, immune score, and ESTIMATE core calculated using the Estimation of STromal and Immune cells in MAlignant Tumor tissues using Expression data (ESTIMATE) algorithm across the LUAD clusters. (**D**) Boxplots of immune exhaustion, immune suppression–related pathway scores across LUAD clusters (C1–C4). (**E**) Boxplots showing immune exclusion–related pathway scores across LUAD clusters (C1–C4) clusters. Statistical significance levels are indicated as follows, ns, no significance; *p < 0.05, **p < 0.01, ***p < 0.001, ****p < 0.0001.

Building on the observed differences in somatic mutation patterns and recognizing that these alterations can influence antitumor immunity. We next explored the tumor immune microenvironment (TME) in LUAD stratified by GRG-based clusters. The expression of key immune checkpoint molecules, including PDCD1 (PD-1), CD274 (PD-L1), CTLA4, LAG3, TIGIT, and HAVCR2 (TIM-3), exhibited heterogeneous patterns across clusters (Fig. 3A). While several immune checkpoint genes showed variation at the transcriptomic level among clusters, no consistent or statistically significant differences in CD274 and HAVCR2, expressions were observed between Clusters C1 and C4. Accordingly, immune checkpoint expression differences were interpreted at a global and pathway level rather than relying on individual checkpoint genes. Previous studies have shown that N-linked glycosylation can stabilize PD-L1 protein and modulate immune checkpoint function [11,46], thus, the observed transcriptional heterogeneity of immune checkpoint–related genes across clusters is compatible with, but does not directly demonstrate, glycosylation-mediated immune regulation. Immune cell infiltration analysis demonstrated notable differences in immune cell composition across clusters (Fig. 3B). Cluster C4 showed relative enrichment of immunosuppressive cell populations such as regulatory T cells (Tregs), plasmacytoid dendritic cells, and exhausted CD8^+^ T cells, whereas Cluster C1 showed relatively higher infiltration of innate immune populations such as neutrophils and natural killer (NK) T cells. Tumor microenvironment characteristics estimated using the ESTIMATE algorithm also varied across clusters. Stromal Score, Immune Score, and ESTIMATE Score differed significantly among clusters (Fig. 3C), although the magnitude of differences between Clusters C1, C2, and C4 was modest. These results were therefore interpreted as reflecting overall differences in TME composition rather than uniform immune enrichment within a single cluster. Further pathway-level analyses indicated that Cluster C4 was enriched for immune-related programs associated with immune exhaustion and immune suppression, including PD-1 signaling, T-cell dysfunction, and myeloid-derived suppressor cell (MDSC) associated signatures (Fig. 3D,E). In parallel, pathways associated with immune exclusion, such as epithelial–mesenchymal transition (EMT), WNT/β-catenin signaling, and TGF-β activity, were preferentially activated in C4. These findings suggest that glycosylation-associated transcriptional programs may contribute to the establishment of a suppressive and exclusionary tumor microenvironment, although these associations remain correlative. By contrast, Cluster C1 exhibited comparatively lower enrichment of immunosuppressive signatures and immune exclusion pathways, suggesting a relatively less suppressive immune contexture. Rather than implying direct therapeutic responsiveness, these patterns suggest that immune-related differences among GRG-defined clusters may influence tumor–immune interactions in a context-dependent manner. Collectively, these results highlight that GRG-defined LUAD clusters capture profound meaningful heterogeneity in immune landscape composition and immune-associated pathway activity. Cluster C4 represents an immunologically suppressed and exclusion-prone subtype at the pathway level, whereas Cluster C1 displays a comparatively less suppressive immune profile. These findings support a role for glycosylation-associated molecular programs in shaping TME heterogeneity and provide a rationale for future studies integrating glycosylation biology with immuno-oncology.

### 3.4 Construction of a Glycosylation Scoring System to Quantify Glycosylation Features in LUAD

**Figure 4:**
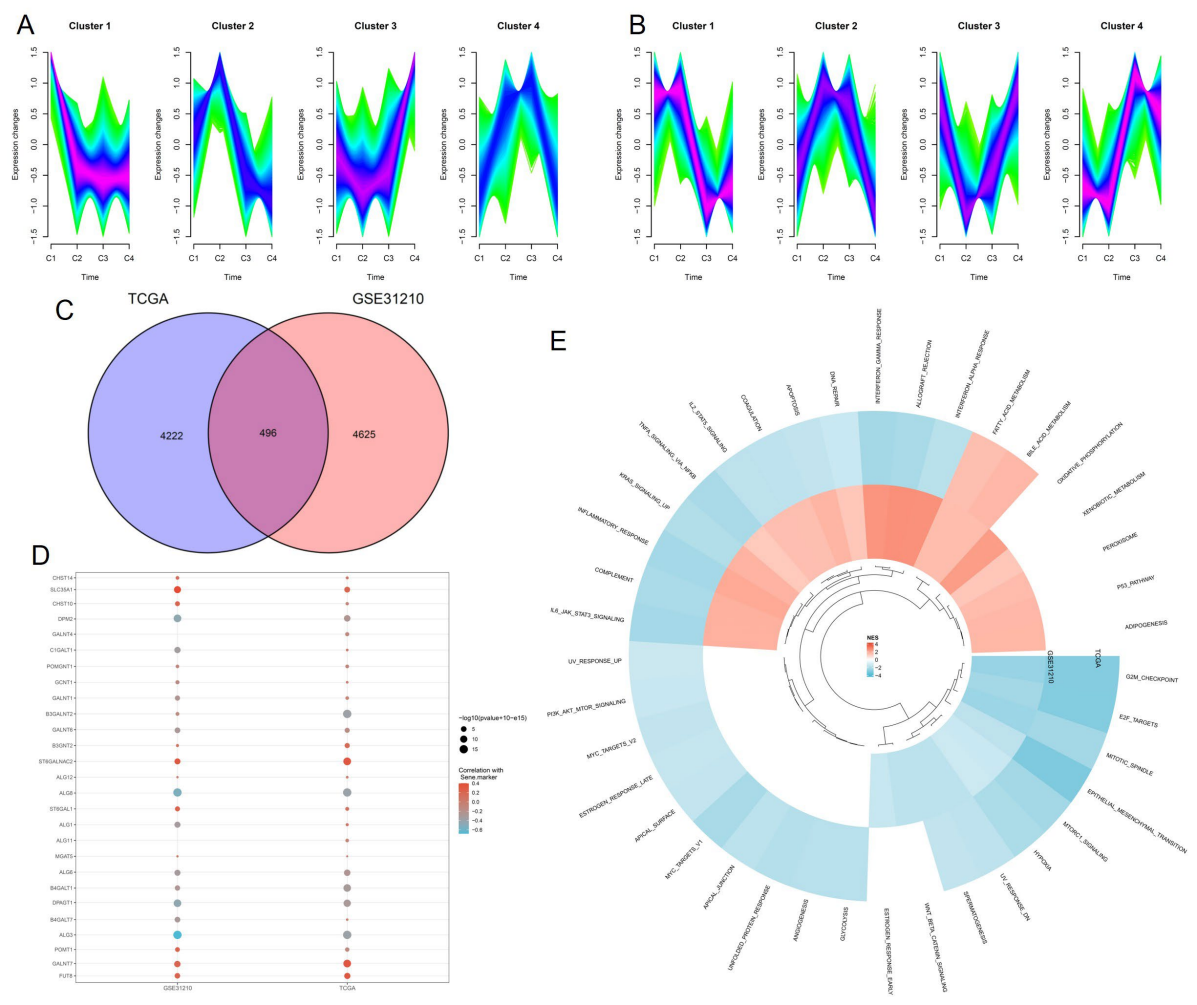
**Identification and validation of glycosylation-related gene (GRG)-based signatures in LUAD.** (**A**) Fuzzy c-means clustering showing four major expression patterns of GRGs in TCGA-LUAD cohort. (**B**) Fuzzy c-means clustering showing four major GRG expression patterns in Gene Expression Omnibus (GEO) dataset GSE31210. (**C**) Venn diagram showing overlap of GRGs identified in TCGA-LUAD and GSE31210 datasets. (**D**) Dot plot displaying correlations between selected glycosylation markers and glycosylation score across cohorts. (**E**) Circular heatmap showing gene set enrichment analysis (GSEA)-based enrichment of glycosylation-related pathways between Glyco.High and Glyco.Low groups.

To quantify the glycosylation status of LUAD patients, we applied fuzzy c-means clustering to GRG expression profiles in TCGA and GSE31210 datasets. Both datasets revealed (Fig. 4A,B), consistent clustering patterns, with distinct temporal expression trajectories across the four LUAD subtypes, highlighting the reproducibility of glycosylation-driven heterogeneity. By intersecting GRGs from TCGA and GSE31210, we identified 496 overlapping genes that were highly conserved between the two cohorts (Fig. 4C) underscoring their robust biological significance. 

From this integrative approach, we extracted a 100-gene panel termed Glyco. marker, which demonstrated strong discriminatory power in distinguishing glycosylation clusters. Correlation analysis revealed that expression of core enzymes such as MGAT5, ST6GAL1, B3GNT2, GALNT7, and FUT8 was positively associated with higher glycosylation scores, whereas ALG family members and certain chaperones related genes showed inverse correlations (Fig. 4D). These results suggest that tumor-promoting glycosylation pathways dominate in Glyco.High groups. Notably, patients in Glyco.High clusters also displayed elevated expression of glycan-modifying enzymes that enhance receptor stabilization and pro-tumorigenic signaling, consistent with prior evidence linking complex N-glycan branching and hyper sialylation to LUAD aggressiveness. Pathway enrichment analysis further demonstrated that the Glyco.marker signature was significantly enriched in tumor-promoting processes such as PI3K/AKT/mTOR signaling, KRAS signaling, epithelial-mesenchymal transition (EMT), and TGF-β pathway activation in Glyco.High tumors (Fig. 4E). Conversely, Glyco.Low groups showed enrichment in DNA repair, apoptosis, and interferon-related immune response pathways, suggesting a less malignant phenotype. Importantly, several immune-related pathways-including antigen processing and checkpoint signaling-were dysregulated in Glyco. High patients, supporting the hypothesis that glycosylation reprogramming may directly regulate immune escape. Overall, these findings establish Glyco. marker as a robust and clinically relevant signature capable of quantifying glycosylation features in LUAD. This scoring system provides a framework to stratify patients by glycosylation activity at both bulk and single-cell resolution, offering a potential biomarker for prognosis, therapeutic response prediction, and mechanistic exploration of glyco-immune interactions.

### 3.5 Association between Glycosylation Features and Stemness in LUAD

**Figure 5:**
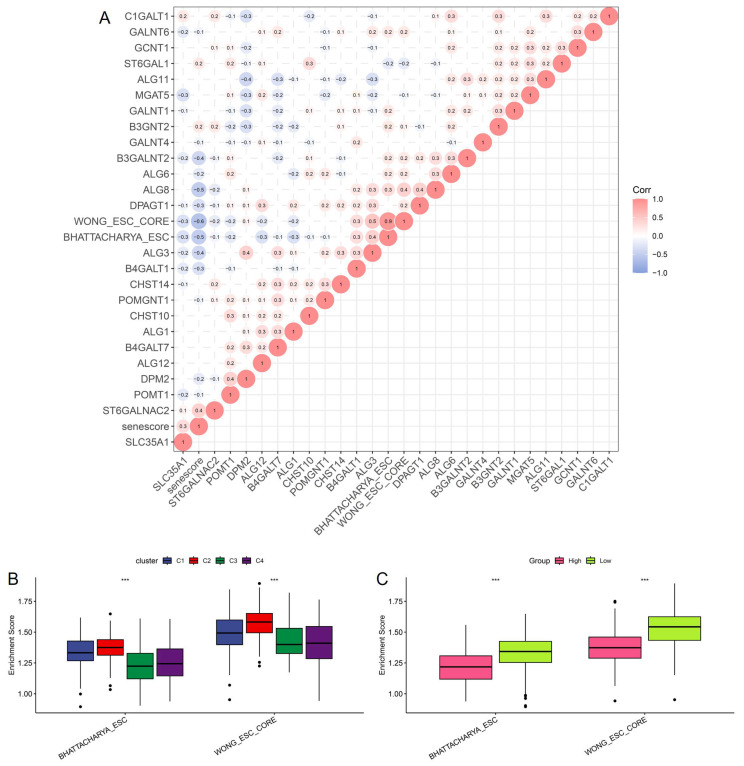
**Glycosylation and stemness association in LUAD.** (**A**) Correlation heatmap between representative GRGs and stemness-related signatures, with positive correlations highlighted in red. (**B**) Boxplots showing enrichment of embryonic stem cell–like signatures, including BHATTACHARYA embryonic stem cell signature (BHATTACHARYA_ESC), and WONG embryonic stem cell core signature (WONG_ESC_CORE) across four glycosylation clusters. (**C**) Comparison of stemness enrichment scores between Glyco.High and Glyco.Low groups. ***p < 0.001.

Given increasing evidence that aberrant glycosylation enhances cellular plasticity, we next investigated the association between glycosylation status and stemness features in LUAD. Correlation analysis revealed that multiple key glycosyltransferases, including MGAT5, ST6GAL1, B3GNT2, and GALNT family members, were strongly and positively correlated with stemness-related signatures. These enzymes are known to regulate N-glycan branching and O-glycan elongation, both of which stabilize cell-surface receptors such as EGFR and CD44, thereby reinforcing stem-like phenotypes and therapy resistance (Fig. 5A). In contrast, ALG family members (ALG1, ALG6, ALG12), which participate mainly in early glycan assembly, exhibited weaker correlations.

We further evaluated stemness enrichment across GRG-defined clusters. Embryonic stem cell–like signatures (BHATTACHARYA_ESC and WONG_ESC_CORE) differed significantly among clusters, with Cluster C4 showing the highest enrichment, followed by C3, while C1 and C2 demonstrated lower enrichment levels (Fig. 5B). When patients were stratified using the glycosylation scoring system, Glyco. High tumors displayed significantly higher stemness scores than Glyco.Low tumors (Fig. 5C).

These observations suggest that LUAD cases with elevated glycosylation activity tend to be associated with stronger stemness programs. Consistent with previous literature, enhanced sialylation and increased β1,6-branching of N-glycans have been reported to stabilize surface receptors such as CD44 and EpCAM and to modulate self-renewal signaling pathways including WNT, NOTCH and PI3K/AKT. Our findings provide correlative support for a link between glycosylation patterns and stemness-associated transcriptional programs in LUAD. Taken together, these results suggest a close association between glycosylation status and stemness phenotypes. Integration of glycosylation scoring with stemness assessment may help identify LUAD patients with more aggressive and therapy-tolerant tumor biology, although functional validation will be required to establish causality. 

### 3.6 Prediction of Potential Therapeutic Agents for GRG-Defined LUAD Subgroups

**Figure 6:**
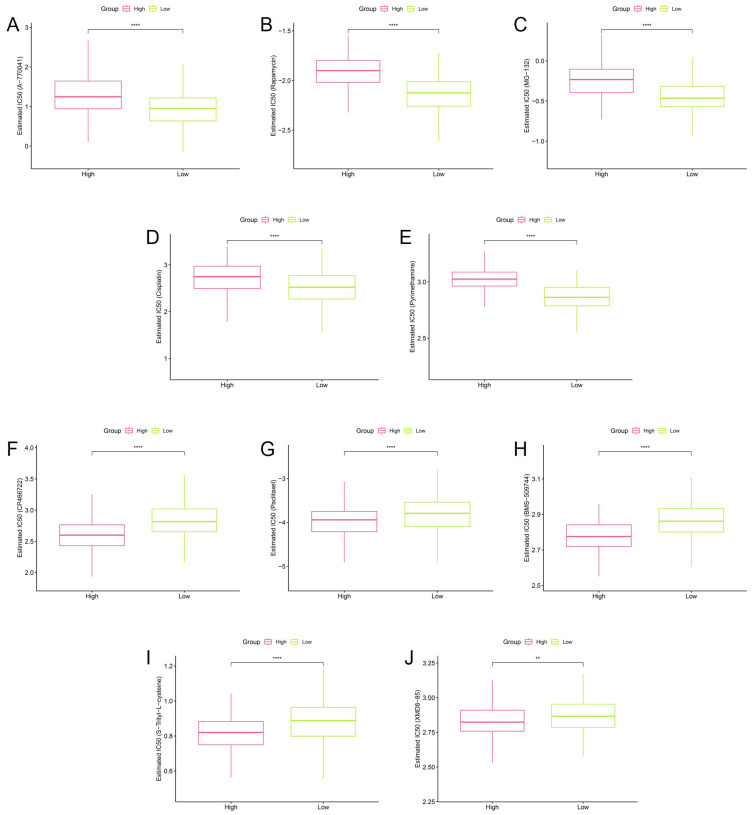
**Drug sensitivity analysis based on the glycosylation scoring system in LUAD.** (**A**–**J**) Boxplots showing the *predicted* half-maximal inhibitory concentration (IC50) values between Glyco.High and Glyco.Low LUAD groups for selected therapeutic agents (**A**) A-770041, (**B**) rapamycin, (**C**) MG-132, (**D**) cisplatin, (**E**) pyrimethamine, (**F**) CP-466722, (**G**) paclitaxel, (**H**) BMS-509744, (**I**) S-trityl-L-cysteine, and (**J**) S-trityl-L-cysteine. Predicted drug sensitivity values were estimated using the OncoPredict algorithm based on the pharmacogenomic models trained on the Genomics of Drug Sensitivity in Cancer (GDSC) dataset. Statistical comparisons between Glyco.High and Glyco.Low groups were performed using the Wilcoxon rank-sum test. Significance levels are indicated as follows: **p < 0.01, ****p < 0.0001.

To evaluate the therapeutic relevance of glycosylation-based classification, we applied the OncoPredict algorithm using Genomics of Drug Sensitivity in Cancer (GDSC) database profiles (Fig. 6A–J). Comparative drug sensitivity analysis revealed that patients in Glyco.High versus Glyco.Low groups exhibited distinct pharmacological responses across multiple classes of agents. Notably, Glyco.High patients showed potentially reduced predicted sensitivity to first-line LUAD chemotherapeutics such as Cisplatin and Pemetrexed. These trends are consistent with prior observation that aberrant glycosylation can be associated with enhanced DNA repair capacity, drug efflux, and apoptosis evasion. Mechanistically, aberrant N- and O-glycosylation may modulate DNA damage response pathways by stabilizing key repair proteins and promoting checkpoint activation, thereby facilitating recovery from cytotoxic injury. In parallel, altered glycosylation increases the expression and membrane localization of ATP-binding cassette (ABC) transporters such as ABCB1 and ABCC1, enhancing drug efflux and may contribute to multidrug resistance. 

Similarly, predicted reduced sensitivity to targeted agents including Gefitinib and Afatinib was more pronounced in Glyco. High tumors, potentially due to altered receptor tyrosine kinase (RTK) glycosylation that stabilizes EGFR and modulates downstream signaling. In support of this, EGFR contains multiple conserved N-glycosylation sites within its extracellular domain; specific residues such as Asn420 and Asn579 have been shown to regulate receptor folding, membrane stability, and ligand affinity. Experimental studies indicate that aberrant N-glycan branching at these sites can be associated with sustained EGFR activation and diminishes the inhibitory effect of tyrosine kinase inhibitors, providing a mechanistic explanation for reduced Gefitinib and Afatinib sensitivity in Glyco.High tumors [47]. 

Conversely, several compounds demonstrated predicted increased sensitivity in Glyco.High subtypes. For instance, inhibitors targeting the PI3K/AKT/mTOR axis (eKIN001-102, dual PI3K/mTOR inhibitors, and AKT inhibitors) showed lower IC50 values in Glyco. High groups, suggesting that glycosylation-driven LUADs may be particularly dependent on these signaling cascades for survival. Moreover, microtubule-targeting agents such as Paclitaxel and Docetaxel also displayed enhanced activity against Glyco. High tumors, possibly reflecting increased mitotic stress associated with abnormal glycan-mediated receptor clustering. Taken together, these findings indicate that glycosylation status is associated with predicted drug response patterns rather than proven clinical outcomes. These results should therefore be regarded as hypothesis-generating. Clinically, Glyco.High patients may benefit from regimens incorporating PI3K/AKT/mTOR pathway inhibitors or microtubule-targeting agents, whereas Glyco.Low patients may show more favorable responses to conventional chemotherapy and EGFR-targeted agents. Importantly, all drug sensitivity predictions in this study are computational estimates derived from GDSC cell-line models and require validation in prospective cohorts and experimental systems.

### 3.7 Identification of Candidate Glycosylation-Associated Regulators Using Signature-Based Analysis in LUAD

**Figure 7:**
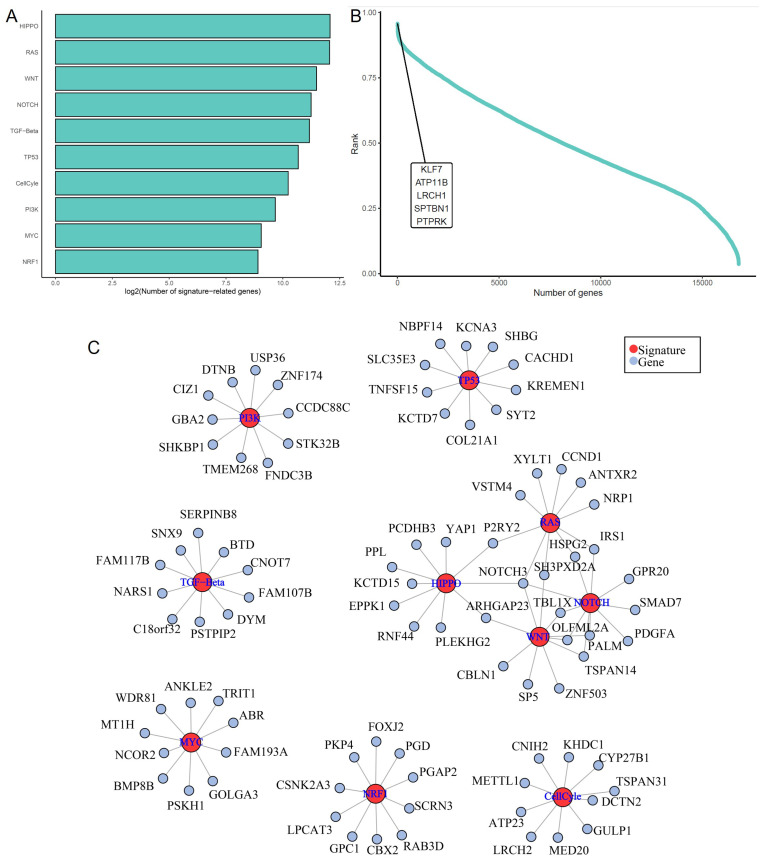
**Identification of new glycosylation-associated marker genes in LUAD using signature-related gene analysis (SRGA).** (**A**) Bar plot showing the number of genes associated with key oncogenic hallmark pathways, Hippo signaling pathway (HIPPO), RAS proto-oncogene signaling (RAS), Wnt signaling pathway (WNT), Notch signaling pathway (NOTCH), transforming growth factor-beta signaling (TGF-β), tumor protein p53 (TP53), phosphoinositide 3-kinase signaling (PI3K), MYC proto-oncogene signaling (MYC), and nuclear factor erythroid 2–related factor signaling (NRF), enriched in glycosylation clusters. (**B**) Ranking curve of candidate genes, prioritized by SRGA, with top-ranked genes including Krüppel-like factor 7 (KLF7), ATPase phospholipid transporting 11B (ATP11B), ucine-rich repeat and calponin homology domain-containing protein 1 (LRCH1), spectrin beta, non-erythrocytic 1 (SPTBN1), and protein tyrosine phosphatase receptor type K (PTPRK) highlighted. (**C**) Network plots showing representative associations between hallmark pathways (red nodes) and identified GRGs (blue nodes).

To identify additional genes associated with glycosylation-driven phenotypes in LUAD, we applied a signature-related gene analysis (SRGA) framework, integrating glycosylation scores, pathway signatures, and co-expression relationships. Pathway enrichment analysis revealed that multiple hallmark oncogenic pathways-including HIPPO, RAS, WNT, NOTCH, and TGF-β were highly connected with GRG expression signatures (Fig. 7A), suggesting close coordination between glycosylation programs and major cancer signaling networks. The ranking analysis identified a set of candidate genes most strong associations to glycosylation scores, among which KLF7, ATP11B, LRCH1, SPTBN1, and PTPRK showed the highest SRGA ranks (Fig. 7B). These genes are not classical glycosyltransferases but have emerging relevance to tumor biology, indicating that glycosylation-associated phenotypes may be influenced by broader regulatory networks beyond canonical enzymatic mediators.

Network visualization further demonstrated interactions between pathway signatures and associated genes (Fig. 7C). Rather than implying direct causation, these networks highlight putative connections between glycosylation status and signaling modules such as RAS, PI3K, NOTCH and TGF-β. These associations should therefore be interpreted as hypothesis-generating links derived from transcriptomic integration, requiring experimental validation in future studies.

Collectively, the SRGA analysis provides a systematic framework for prioritizing novel glycosylation-associated candidate markers beyond canonical enzymes such as MGAT5 or ST6GAL1. The integration of transcriptomic signatures and glycosylation scoring not only broadens the repertoire of potential biomarkers but also highlights previously underappreciated regulatory genes that may serve as therapeutic targets or prognostic indicators in LUAD.

### 3.8 Single-Cell Transcriptomics Reveals Cell Type–Specific Expression Patterns of GRGs in LUAD

**Figure 8:**
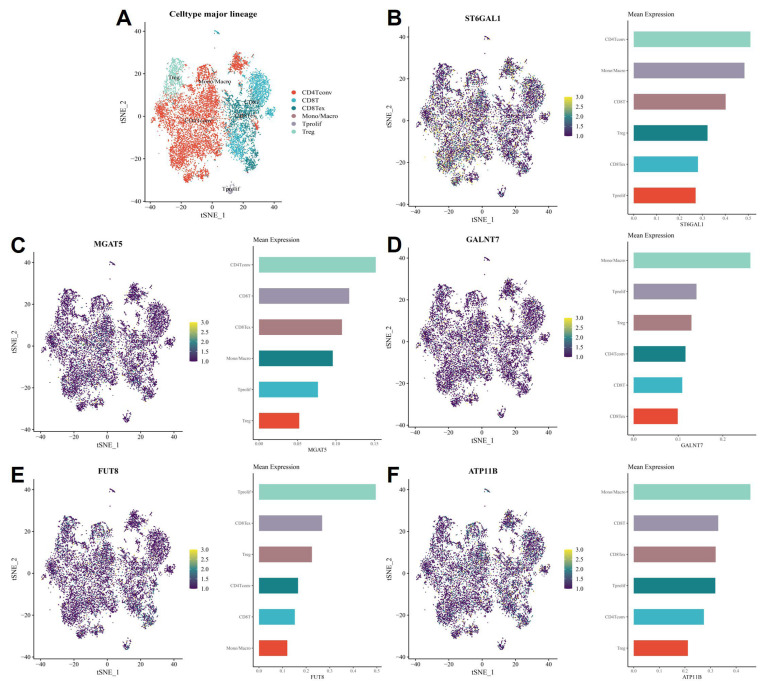
**Single-cell analysis of GRG expression in LUAD.** (**A**) The t-distributed stochastic neighbor embedding (t-SNE) plot of single-cell clustering, where different colors represent distinct cell lineages, including D4^+^ conventional T cells (CD4^+^ Tconv), CD8^+^ T cells, (CD8^+^ T), exhausted CD8^+^ T cells (CD8^+^ Tex), monocytes/macrophages, proliferating T cells (Tprolif), and regulatory T cells (Tregs). (**B**–**F**) t-SNE plots showing the expression distribution of representative GRGs (ST6GAL1 (**B**), MGAT5 (**C**), GALNT7 (**D**), FUT8 (**E**), ATP11B (**F**)) across different cell types. Brighter colors indicate higher expression levels. Bar charts on the right display mean expression levels of these genes in each cell lineage.

To investigate the cellular heterogeneity of glycosylation in LUAD, we performed single-cell RNA sequencing analysis. Unsupervised clustering identified major immune and tumor-associated populations, including CD4^+^ Tconv, CD8^+^ T cells, exhausted CD8^+^ T cells (CD8^+^ Tex), proliferating T cells (Tprolif), regulatory T cells (Tregs), and monocytes/macrophages (Fig. 8A). We then assessed the expression of representative GRGs that were identified as significant in our bulk transcriptome and SRGA analyses. The sialyltransferase ST6GAL1 (Fig. 8B) was broadly expressed across tumor-associated immune cells but showed particularly high levels in CD4^+^ Tconv and monocytes/macrophages, supporting its role in modulating cell–cell interactions and immune suppression. The branching enzyme MGAT5 (Fig. 8C) was enriched in CD8^+^ Tconv and macrophages, consistent with its function in stabilizing glycoproteins such as PD-L1. GALNT7 (Fig. 8D), a regulator of O-glycosylation initiation, exhibited higher expression in macrophages and CD4^+^ Tconv subsets, suggesting involvement in antigen processing and immune regulation. The core fucosyltransferase FUT8 (Fig. 8E) showed strong enrichment in proliferating T cells and exhausted CD8^+^ T cells, indicating potential roles in T cell activation and dysfunction. Notably, ATP11B (Fig. 8F), a novel candidate identified in our SRGA pipeline, was expressed in both tumor-infiltrating macrophages and Tregs, suggesting a dual function in cancer progression and shaping of the immunosuppressive microenvironment. Together, these results demonstrate that glycosylation-related gene expression is not uniform but highly cell type-specific in LUAD. Tumor epithelial cells remain the primary site of glycosylation reprogramming; however, significant expressions in immune subsets such as Tregs and macrophages highlight that glycosylation may also directly shape the tumor immune microenvironment. This single-cell perspective provides mechanistic insight into how glycosylation orchestrates both malignant progression and immune evasion in LUAD.

### 3.9 IHC Validation of Glycosylation Regulators in LUAD

**Figure 9:**
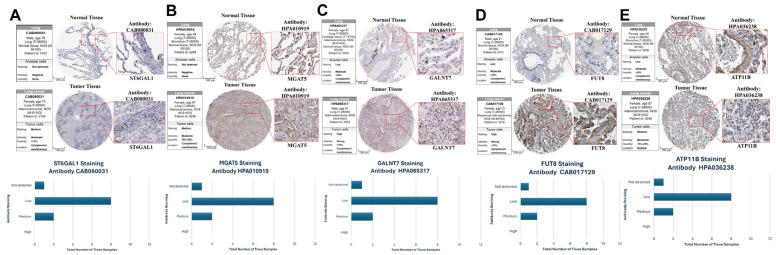
**Immunohistochemical profiling of glycosylation regulators in LUAD.** (**A**–**E**) Representative immunohistochemistry (IHC) images showing protein expression of key glycosylation-related genes (GRGs) in normal lung tissue (upper panels) and LUAD tumor tissue (middle panels) obtained from the Human Protein Atlas (HPA) database. For each marker, low-magnification tissue sections and corresponding zoomed regions highlight staining patterns within epithelial compartments. Antibody identifiers used for staining are indicated below each panel: (**A**) β-galactoside α-2,6-sialyltransferase 1 (ST6GAL1; antibody CAB056031), (**B**) mannosyl (α-1,6)-glycoprotein β-1,6-N-acetylglucosaminyltransferase (MGAT5; antibody HPA070519), (**C**) polypeptide N-acetylgalactosaminyltransferase 7 (GALNT7; antibody HPA066517), (**D**) fucosyltransferase 8 (FUT8; antibody CAB071720), and (**E**) ATPase phospholipid transporting 11B (ATP11B; antibody HPA063628). The lower panels summarize cohort-level staining distributions across LUAD samples based on the Human Protein Atlas scoring system (high, medium, low, or not detected).

To evaluate protein-level expression of key glycosylation-related regulators in LUAD, immunohistochemistry (IHC) images were retrieved from the Human Protein Atlas (HPA) database (Fig. 9A–E). Representative staining patterns in normal lung tissue and LUAD tumor tissue were examined for β-galactoside α-2,6-sialyltransferase 1 (ST6GAL1), mannosyl (α-1,6)-glycoprotein β-1,6-N-acetylglucosaminyltransferase (MGAT5), polypeptide N-acetylgalactosaminyltransferase 7 (GALNT7), fucosyltransferase 8 (FUT8), and ATPase phospholipid transporting 11B (ATP11B).

Overall, these markers exhibited heterogeneous protein expression patterns across LUAD samples rather than uniform overexpression. ST6GAL1 showed predominantly weak or undetectable staining in both normal and tumor tissues (Fig. 9A). In contrast, MGAT5 displayed moderate epithelial staining in tumor tissues compared with normal lung samples (Fig. 9B). GALNT7 demonstrated variable cytoplasmic staining intensity across tumor specimens, with a subset of cases showing stronger expression (Fig. 9C). FUT8 showed moderate cytoplasmic staining in malignant epithelial cells (Fig. 9D), while ATP11B exhibited heterogeneous expressions were ranging from weak to moderate cytoplasmic or membranous staining among tumor samples (Fig. 9E). Taken together, these IHC observations highlight substantial intertumoral variability in the protein expression of glycosylation regulators in LUAD. The observed staining patterns are broadly consistent with the transcriptomic heterogeneity identified in the GRG-based analyses, while also underscoring that mRNA-protein concordance is not always direct. These results therefore provide supportive protein-level evidence for the involvement of glycosylation regulators in LUAD biology. 

## 4 Discussion

Recent studies have demonstrated that tumor glycosylation is highly heterogeneous, exhibiting both intertumoral and intratumoral variability associated with genetic alterations, metabolic states, and microenvironmental cues [48,49]. In LUAD, dynamic changes in glycosylation patterns across tumor regions and disease stages may influence signaling activity, immune interactions, and therapeutic response. Therefore, glycosylation-associated molecular features should be interpreted as context-dependent programs rather than fixed tumor properties. In this study, we systematically characterized GRGs in LUAD and identified four robust molecular subtypes with distinct clinicopathological features, genomic alterations, immune landscapes, and therapeutic responses. By integrating multi-database resources and multi-omics analyses, we not only established a glycosylation scoring system (Glyco.marker) but also uncovered novel GRG-associated candidates through SRGA, thereby providing new insights into the biological and clinical significance of glycosylation in LUAD.

Our findings highlight the pivotal role of aberrant glycosylation in LUAD pathogenesis. GRG-based clustering clearly separated LUAD patients into subgroups with different prognoses, with Cluster C4 showing the poorest survival. This subgroup was enriched in upregulation of enzymes such as MGAT5, ST6GAL1, and GALNTs, known drivers of complex N-glycan branching and hyper sialylation, while Cluster C1 retained a relatively balanced glycosylation pattern. These data support the view that aberrant glycosylation reprogramming enhances tumor aggressiveness and contributes to worse outcomes [7,50,51,52].

At the genomic level, distinct mutational profiles emerged across GRG-defined clusters. Enrichment of TP53 mutations in *Glyco.High* tumors suggest an association between impaired genomic surveillance and glycosylation-associated malignant progression. Co-mutation patterns such as KRAS/STK11 and KEAP1/NFE2L2 further suggest interplay between glycosylation, redox regulation, and RTK signaling, which may contribute to oncogenic dependencies in LUAD [27,53]. Emerging evidence suggests that dysregulation of the KEAP1/NFE2L2 axis may provide a mechanistic bridge between oxidative stress and aberrant glycosylation. Activation of NFE2L2 rewires cellular metabolism and increases flux through the hexosamine biosynthetic pathway, raising intracellular UDP-GlcNAc levels and thereby supporting complex N-glycan branching mediated by enzymes such as MGAT5. Oxidative stress also modifies ER and Golgi redox balance, affecting protein folding, glycoprotein quality control, and the activity of multiple glycosyltransferases and sialyltransferases. Through these mechanisms, redox imbalance may be associated with pro-tumorigenic glycosylation patterns, stabilize receptor tyrosine kinases and immune checkpoint molecules, and possibly immune evasion and therapy resistance in LUAD [54]. Somatic mutations such as TP53, KRAS, and MUC16 may indirectly influence glycosylation through transcriptional regulation or altered oncogenic signaling pathways rather than direct causal mechanisms. In addition to single-gene mutations, chromosomal context may also contribute to glycosylation remodeling in LUAD. The chromosomal regions 19q13, which harbor several FUT family members, and 12q13, which contains multiple GALNT genes, are frequently altered in LUAD. These loci show recurrent copy number gains and segmental amplifications in public LUAD cohorts, suggesting that structural genomic instability may be associated with increased dosage of glycosyltransferase genes. Such copy number variation provides a plausible mechanism for the simultaneous deregulation of multiple glycosylation enzymes and supports a link between regional chromosomal alterations and glycosylation reprogramming. The prominence of MUC16 alterations in Cluster C3 may also have immunologic implications. MUC16 is a heavily glycosylated mucin bearing abundant O- and N-glycan structures, and its aberrant glycosylation has been linked to modulation of antitumor immunity. Experimental studies have shown that MUC16 can impair natural killer (NK) cell recognition by masking activating ligands and engaging inhibitory receptors, and that MUC16-associated glycans influence macrophage signaling and polarization toward immunosuppressive phenotypes [55,56]. These observations support the possibility that MUC16 mutation and dysregulated glycosylation in Cluster C3 may be associated with glycan-mediated immune evasion in LUAD. Consistent with this, our co-mutation analysis suggests that these alterations converge on pathways related to oxidative stress adaptation, PI3K/AKT signaling, epithelial–mesenchymal transition, and immune regulation, which are closely linked to glycosylation remodeling in LUAD.

The tumor immune microenvironment (TME) also displayed striking heterogeneity across GRG subtypes. Cluster C4 exhibited elevated expression of immune checkpoint molecules (PD-1, PD-L1, CTLA-4, LAG3, TIGIT) alongside increased infiltration of immunosuppressive cells such as Tregs and plasmacytoid dendritic cells. These findings are consistent with the role of N-linked glycosylation in stabilizing PD-L1 and are associated with immune evasive phenotypes. Conversely, Glyco.Low tumors were more permissive to immune infiltration and may represent immunologically “hotter” subtypes with greater potential responsiveness to immunotherapy [57,58]. To quantify glycosylation heterogeneity, we constructed the Glyco. marker scoring system. This 100-gene panel effectively stratified LUAD patients into Glyco. High and Glyco.Low groups, with the Glyco.High phenotype enriched for oncogenic pathways such as PI3K/AKT/mTOR, KRAS signaling, EMT, and TGF-β. By contrast, Glyco.Low tumors showed enrichment in apoptosis and interferon response pathways, suggesting a less aggressive molecular phenotype [27,52].

Functional characterization revealed a strong association between glycosylation and stemness. Glyco.High tumors exhibited enrichment of embryonic stem cell–like signatures and positive correlations with stemness regulators (CD44, EpCAM, KLF4). These findings suggest that aberrant glycan modifications may be linked to stabilizing self-renewal pathways and enhance tumor cell plasticity, which in turn may be associated with recurrence and therapeutic resistance [59,60]. Therapeutic prediction analyses further indicated that glycosylation status is associated with differences in predicted drug response (Fig. 5). Glyco. High tumors were resistant to conventional platinum-based chemotherapy and EGFR tyrosine kinase inhibitors yet showed increased sensitivity to PI3K/AKT/mTOR inhibitors and microtubule-targeting agents such as paclitaxel and docetaxel. The increased activity of Paclitaxel and Docetaxel in Glyco.High tumors were identified through OncoPredict-based computational predictions and should be considered hypothesis-generating. Aberrant glycosylation has been reported to influence centrosome function, spindle organization, and microtubule stability, which may increase mitotic stress and render highly glycosylated tumors more susceptible to microtubule-targeting agents. The enhanced sensitivity of Glyco.High tumors to PI3K/AKT/mTOR inhibition is mechanistically plausible. Aberrant N-glycan branching can stabilize upstream receptor tyrosine kinases such as EGFR and IGF1R at the plasma membrane, sustaining PI3K/AKT signaling. In parallel, increased flux through the hexosamine biosynthetic pathway in highly glycosylated tumors raises UDP-GlcNAc levels, promoting complex N-glycosylation and creating a positive feedback loop between glycosylation and PI3K pathway activation. Through these mechanisms, Glyco.High LUADs may become increasingly dependent on PI3K/AKT/mTOR signaling, which provides a rationale for their selective vulnerability to inhibitors targeting this axis. By contrast, Glyco.Low patients retained sensitivity to standard chemotherapies, consistent with their more favorable clinical outcomes. These observations underscore the translational potential of GRG profiling for guiding precision therapy [20,61,62]. Importantly, the application of signature-related gene analysis (SRGA) identified novel GRG-associated markers, including ATP11B, KLF7, PTPRK, and LRCH1, many of which are connected with key oncogenic pathways such as RAS, PI3K, and TGF-β [63,64,65]. These candidates, though not traditionally categorized as glycosylated enzymes, may represent indirect regulators of glyco-phenotypes in LUAD and warrant further experimental validation [50].

Finally, our integration of single-cell transcriptomic data further revealed that GRGs are expressed in a cell type-specific manner, with tumor epithelial cells showing the highest abundance of MGAT5 and ST6GAL1, while immune subsets such as Tregs and macrophages also displayed substantial expressions of GALNT7, FUT8, and ATP11B. These findings indicate that glycosylation reprogramming is not restricted to tumor-intrinsic processes but extends to the tumor immune microenvironment, where it may promote immunosuppressive activity and therapy resistance. From a translation perspective, this single-cell evidence highlights the potential value of combining glycosylation-targeted interventions with immunotherapy, as both malignant and immune compartments may contribute to glycan-mediated immune evasion [50]. 

Looking forward, integrative multi-omics approaches will be essential to validate the interplay between glycosylation remodeling and mutational architecture in LUAD. Combining genomic and transcriptomic profiling with mass-spectrometry–based glycoproteomics could directly assess how specific driver mutations reshape glycan structures at the protein level. Single-cell multi-omics and spatially resolved technologies may further clarify how these relationships vary across tumor subclones and the immune microenvironment. Such efforts will help translate GRG-defined subtypes into clinically actionable biomarkers and guide the development of glycosylation-targeted therapeutic strategies. Taken together, our results demonstrate that GRG-defined LUAD subtypes capture essential aspects of tumor biology, including survival heterogeneity, mutational architecture, immune evasion, stemness plasticity, and therapy response [20,66]. The Glyco marker scoring system and SRGA-derived candidates not only expand the repertoire of prognostic biomarkers but also highlight potential therapeutic vulnerabilities. Future investigations combining glycomics, proteomics, and functional studies are required to validate these findings and explore the feasibility of integrating glycosylation-targeted interventions with immunotherapy and pathway inhibitors. Also, from a translational perspective, our results suggest that glycosylation signatures may be developed as companion biomarkers to guide treatment selection. In particular, patients with Glyco.High tumors could be prospectively enriched in clinical trials evaluating PI3K/AKT/mTOR inhibitors or microtubule-targeting agents, whereas Glyco.Low tumors may remain candidates for standard chemotherapy or EGFR-targeted therapy. Integration of glycosylation scoring into patient-stratification frameworks may therefore help refine precision-medicine strategies in LUAD. In parallel, direct targeting of aberrant glycosylation itself represents an emerging therapeutic strategy. Prototype compounds include tunicamycin analogs that inhibit N-linked glycosylation, fucosyltransferase inhibitors that reduce core fucosylation, and O-GlcNAc transferase (OGT) inhibitors that block O-GlcNAc cycling. Although most of these agents are still at the preclinical or early clinical-development stage, they highlight the feasibility of exploiting glycan-dependent vulnerabilities in LUAD and support further investigation of glycosylation-directed therapies.

## 5 Conclusions

This study provides a comprehensive characterization of GRGs in LUAD, revealing distinct molecular subtypes that differ in prognosis, genomic architecture, immune microenvironment, and therapeutic responsiveness. We established a glycosylation scoring system (Glyco.marker) to quantify glycosylation activity, which effectively stratified patients into prognostic groups and identified treatment vulnerabilities, particularly in the context of PI3K/AKT/mTOR signaling and microtubule-targeting agents. Novel GRG-associated markers identified by SRGA further broaden the spectrum of potential biomarkers and therapeutic targets.

**Figure 10:**
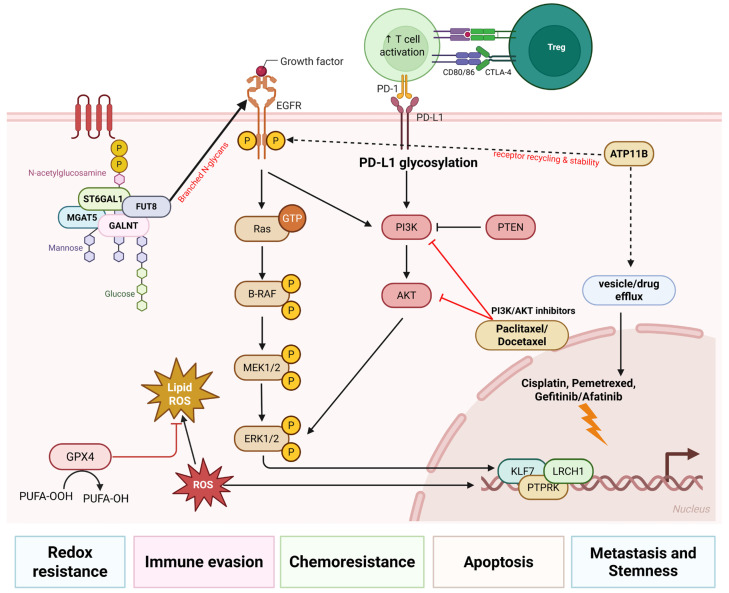
**Glycosylation rewires oncogenic signaling, immune evasion, and drug response in LUAD.** N- and O-glycosylation enzymes (MGAT5, ST6GAL1, FUT8, GALNT family) are associated with modulation of EGFR and PD-L1 signaling. Glycosylation is linked to activation of downstream pathways including RAS–RAF–MEK–ERK and PI3K–AKT, as well as regulation of redox balance and immune suppression. Stabilization of PD-L1 is associated with reduced T-cell activity and enrichment of immunosuppressive phenotypes. These processes are further connected to drug response patterns, including reduced sensitivity to conventional therapies and potential vulnerability to PI3K/AKT inhibitors and microtubule-targeting agents. Additional regulatory genes (KLF7, LRCH1, PTPRK) are associated with apoptosis and stemness-related programs. Arrows indicate activation, T-bars indicate inhibition, and dashed lines represent trafficking or recycling processes.

The integration of single-cell transcriptomic profiling and immunohistochemical validation not only confirmed the transcriptomic findings but also underscored the cell type–specific and protein-level relevance of glycosylation reprogramming. Together, these results highlight glycosylation as a key determinant of LUAD heterogeneity and progression, offering new opportunities for biomarker-driven precision oncology. Future investigations incorporating multi-omics datasets, functional experiments, and clinical validation are warranted to accelerate the translation of glycosylation-based strategies into practice. Future studies that incorporate multi-omics profiling, experimental validation, and clinical trials will be essential to translate these insights into effective diagnostic and therapeutic strategies (Fig. 10).

## 6 Limitations

Several limitations should be acknowledged in this study. First, our analyses were primarily based on retrospective datasets from TCGA and GEO, which may introduce biases related to patient selection, sequencing platforms, and clinical annotation. Although we employed multiple independent cohorts for validation, prospective studies with well-annotated clinical samples are required to confirm the robustness and clinical utility of our findings. Second, glycosylation is a dynamic and context-dependent post-translational process influenced by cellular metabolism, microenvironmental conditions, and regulatory mechanisms. Accordingly, transcriptomic profiling of glycosylation-related genes (GRGs) provides an indirect representation of glycosylation-associated molecular programs and does not fully capture glycan structures or their functional consequences. Integration of proteomic and glycomic approaches will therefore be essential in future studies to directly characterize glycosylation modifications. Third, although the glycosylation-related scoring system and the SRGA framework enabled identification of candidate GRG-associated markers, their mechanistic roles in LUAD progression remain to be experimentally validated. Functional assays, including gene perturbation, modulation of glycosylation pathways, and *in vivo* models, will be necessary to establish causal relationships. Fourth, although we explored drug sensitivity predictions, these analyses were computational in nature and required pharmacological validation in preclinical and clinical settings. Finally, LUAD is a highly heterogeneous disease influenced by factors such as smoking history, genetic background, and immune contexture, which may affect the generalizability of our findings across patient populations. Future studies integrating multi-omics data, functional experiments, and prospective clinical validation will be important to translate glycosylation-associated biomarkers and therapeutic insights into clinical practice.

## Data Availability

All data involved in this study are available from the corresponding author upon reasonable request. Publicly available data sets were used in the analyses. Transcriptomic and clinical data for LUAD were obtained from The Cancer Genome Atlas (TCGA) (https://portal.gdc.cancer.gov/) and the Gene Expression Omnibus (GEO) (https://www.ncbi.nlm.nih.gov/geo/). Glycosylation-related gene sets were curated from the Molecular Signatures Database (MSigDB) (https://www.gsea-msigdb.org/gsea/msigdb/human/collections.jsp), Enrichr (https://maayanlab.cloud/Enrichr/), Harmonizome 3.0 (https://maayanlab.cloud/Harmonizome/), and the GlycoGene Database (https://acgg.asia/db/glycodb/).

## Supplementary Materials

The supplementary material is available online at https://www.techscience.com/doi/10.32604/or.2026.074013/s1.

## Abbreviations

LUAD	Lung adenocarcinoma
NSCLC	Non-small cell lung cancer
GRGs	Glycosylation-related genes
TCGA	The Cancer Genome Atlas
GEO	Gene Expression Omnibus
TPM	Transcripts per kilobase million
TP53	Tumor protein p53
EGFR	Epidermal growth factor receptor
TKI	Tyrosine kinase inhibitor
CNV	Copy number variation
TMB	Tumor mutation burden
MATH	Mutant-allele tumor heterogeneity
TME	Tumor microenvironment
ssGSEA	Single-sample gene set enrichment analysis
GSVA	Gene set variation analysis
ESTIMATE	Estimation of STromal and Immune cells in MAlignant Tumor tissues using Expression data
IOBR	Immuno-oncology biological research
GSEA	Gene set enrichment analysis
GDSC	Genomics of Drug Sensitivity in Cancer
SRGA	Signature-related gene analysis
IC50	Half-maximal inhibitory concentration
PI3K	Phosphoinositide 3-kinase
mTOR	Mammalian target of rapamycin
EMT	Epithelial–mesenchymal transition
PD-1	Programmed cell death protein 1
PD-L1	Programmed death-ligand 1
CTLA-4	Cytotoxic T-lymphocyte-associated protein 4
MDSC	Myeloid-derived suppressor cell
Treg	Regulatory T cell
Glyco.High	High glycosylation activity group
Glyco.Low	Low glycosylation activity group
Glyco.marker	Glycosylation-based gene signature scoring system
CD4^+^ Tconv	Conventional CD4^+^ T cell
CD8^+^ T	Cytotoxic CD8^+^ T cell
CD8^+^ Tex	Exhausted CD8^+^ T cell
Tprolif	Proliferating T cell
