# Supplementary material for: Integrative Analysis of Glycosylation-Related Genes Reveals Prognostic Subtypes, Immune Evasion, and Therapeutic Vulnerabilities in Lung Adenocarcinoma
Source: Oncol Res. 2026 Jun 16;34(7):18. doi: 10.32604/or.2026.074013 (PMC13292060; doi:10.32604/or.2026.074013)
Supplement: Supplementary file 1 [file OncolRes-34-74013-s001.zip › TSP_OR_74013-s001.docx]

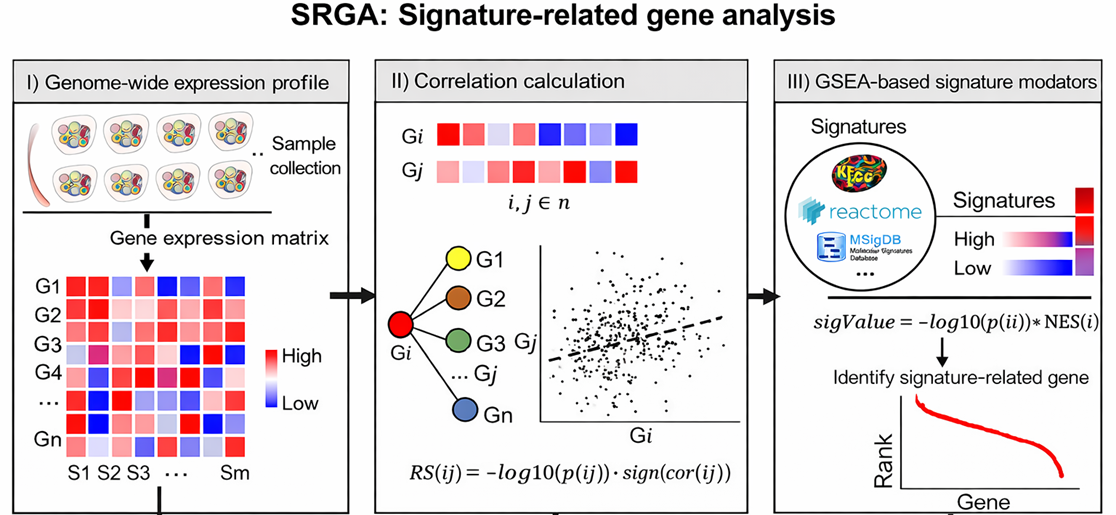


**Supplementary Figure S1. Workflow of signature-related gene analysis (SRGA).**Schematic illustration of the signature-related gene analysis (SRGA) framework used to identify glycosylation-associated candidate regulators. (I) Genome-wide gene expression profiles were obtained from bulk transcriptomic datasets to generate a gene expression matrix across samples. (II) Pairwise correlation analysis was performed between individual genes, and a correlation score was calculated by integrating correlation direction and statistical significance $RS(i,j)=-log10(p(i,j))\times sign(cor(i,j))$. (III) Gene set enrichment analysis (GSEA) was applied using curated pathway and signature databases (e.g., KEGG, Reactome, MSigDB) to identify genes strongly associated with glycosylation-related signatures. Genes were ranked based on their association strength with pathway signatures, and top-ranked genes were prioritized as candidate glycosylation-associated regulators. This framework is designed as an integrative prioritization strategy rather than a causal inference model.


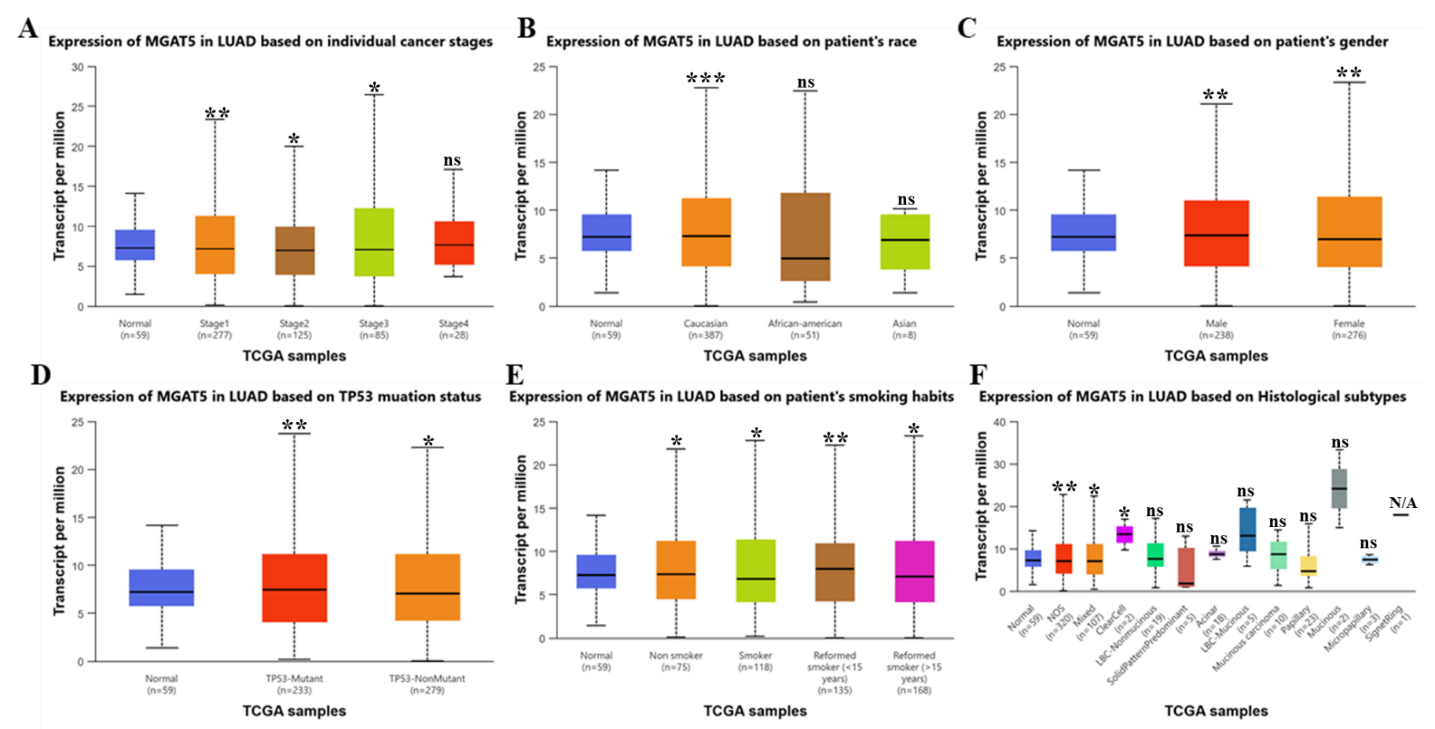
 **Supplementary Figure S2. Clinical stratification of MGAT5 expression in lung adenocarcinoma.** Boxplots showing MGAT5 mRNA expression (transcripts per million, TPM) in lung adenocarcinoma (LUAD) and normal lung tissues using TCGA data accessed via the UALCAN platform.(A) MGAT5 expression stratified by individual tumor stage (Stage I–IV). (B) MGAT5 expression according to patient race. (C) MGAT5 expression according to patient gender. (D) MGAT5 expression based on TP53 mutation status. (E) MGAT5 expression according to patients’ smoking history. (F) MGAT5 e*x*pression across different histological subtypes of LUAD. Statistical significance between groups is indicated as *P < 0.05, **P < 0.01, ***P < 0.001; ns, not significant. N/A indicates comparisons that were not tested due to insufficient sample numbers.

###
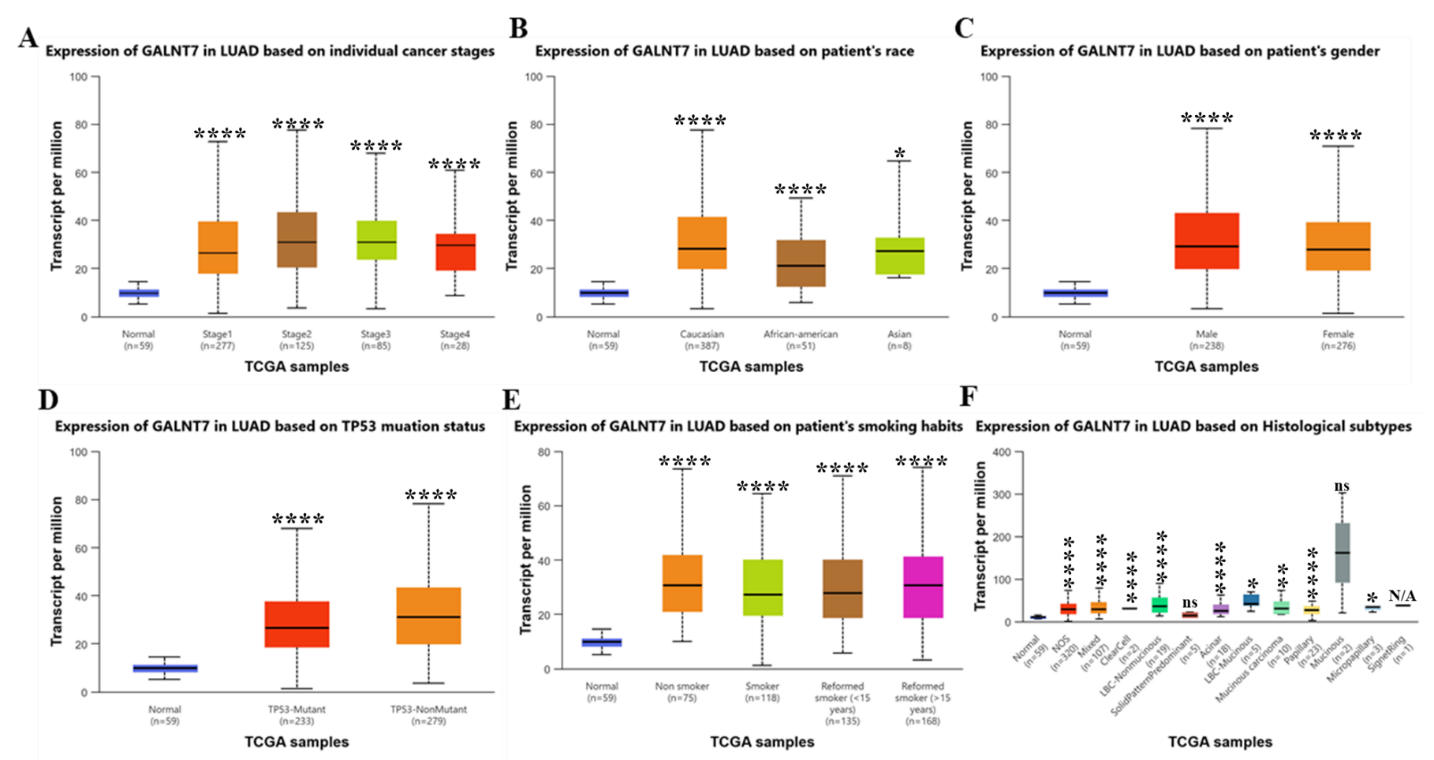
 ****Supplementary Figure S3. Clinical stratification of GALNT7 expression in lung adenocarcinoma.**** Boxplots depicting **GALNT7 mRNA expression** (TPM) in LUAD and normal lung tissues derived from TCGA and analyzed using UALCAN. (A) GALNT7 expression stratified by individual tumor stage (Stage I–IV). (B) GALNT7 expression according to patient race. (C) GALNT7 expression according to patient gender. (D) GALNT7 expression based on TP53 mutation status. (E) GALNT7 expression according to patients’ smoking history. (F) GALNT7 expression across different histological subtypes of LUAD. Statistical significance between groups is indicated as *P < 0.05, **P < 0.01, ****P < 0.0001; ns, not significant. N/A indicates comparisons that were not tested due to insufficient sample numbers.

### ****
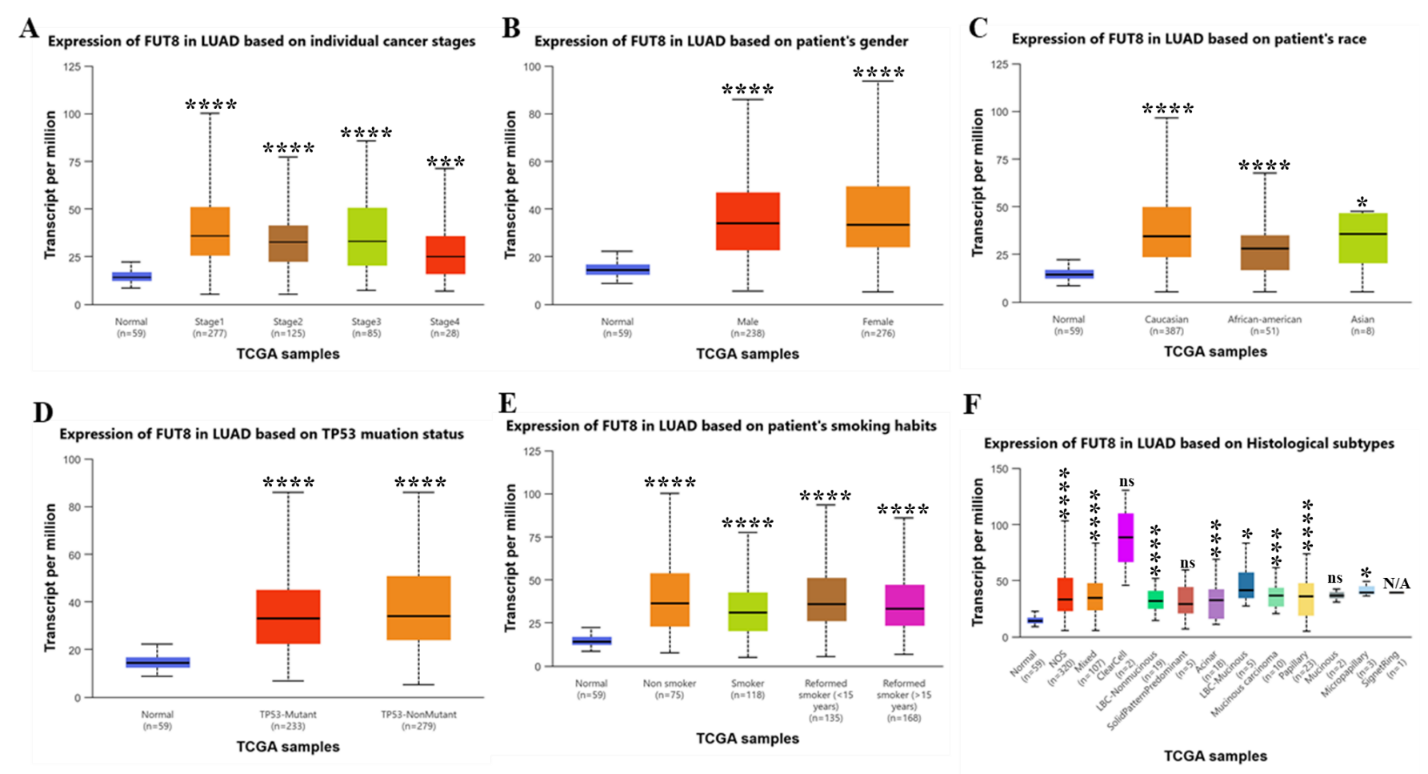
 Supplementary Figure S4. Clinical stratification of FUT8 expression in lung adenocarcinoma.** Boxplots showing FUT8 mRNA expression (transcripts per million, TPM) in lung adenocarcinoma (LUAD) and normal lung tissues using TCGA data accessed through the UALCAN platform. (A) FUT8 expression stratified by individual tumor stage (Stage I–IV). (B) FUT8 expression according to patient race. (C) FUT8 expression according to patient gender. (D) FUT8 expression based on TP53 mutation status. (E) FUT8 expression according to patients’ smoking history. (F) FUT8 expression across different histological subtypes of LUAD.** Statistical significance between groups is indicated as *P < 0.05, ***P < 0.001, ****P < 0.0001; ns, not significant. N/A indicates comparisons that were not tested due to insufficient sample numbers.

###
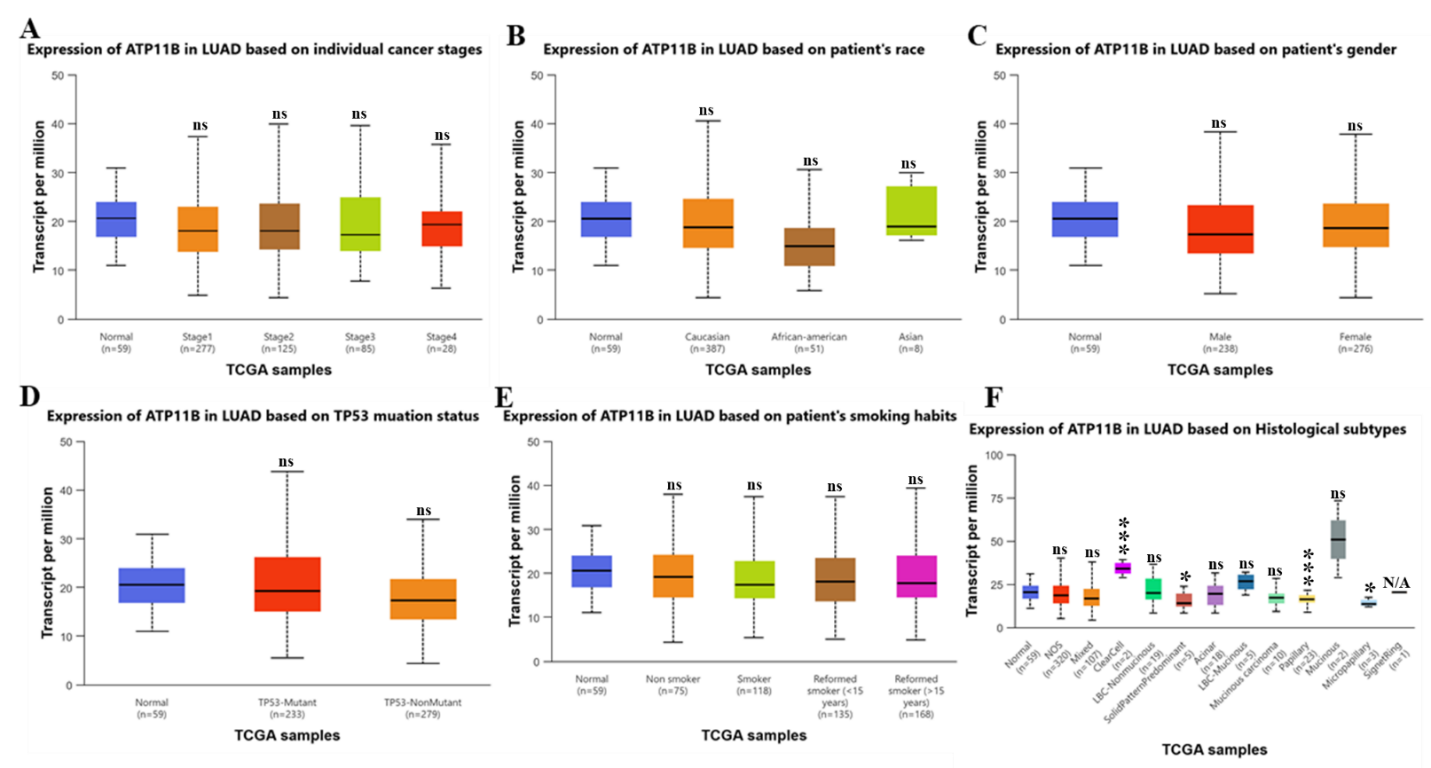
****Supplementary Figure S5. Clinical stratification of ATP11B expression in lung adenocarcinoma.**** Boxplots showing **ATP11B mRNA expression** (TPM) in LUAD and normal lung tissues using TCGA data accessed through UALCAN. (A) ATP11B expression stratified by individual tumor stage (Stage I–IV). (B) ATP11B expression according to patient race. (C) ATP11B expression according to patient gender. (D) ATP11B expression based on TP53 mutation status. (E) ATP11B expression according to patients’ smoking history. (F) ATP11B expression across different histological subtypes of LUAD. Statistical significance between groups is indicated as *P < 0.05, ***P < 0.001; ns, not significant. N/A indicates comparisons that were not tested due to insufficient sample numbers.

### **
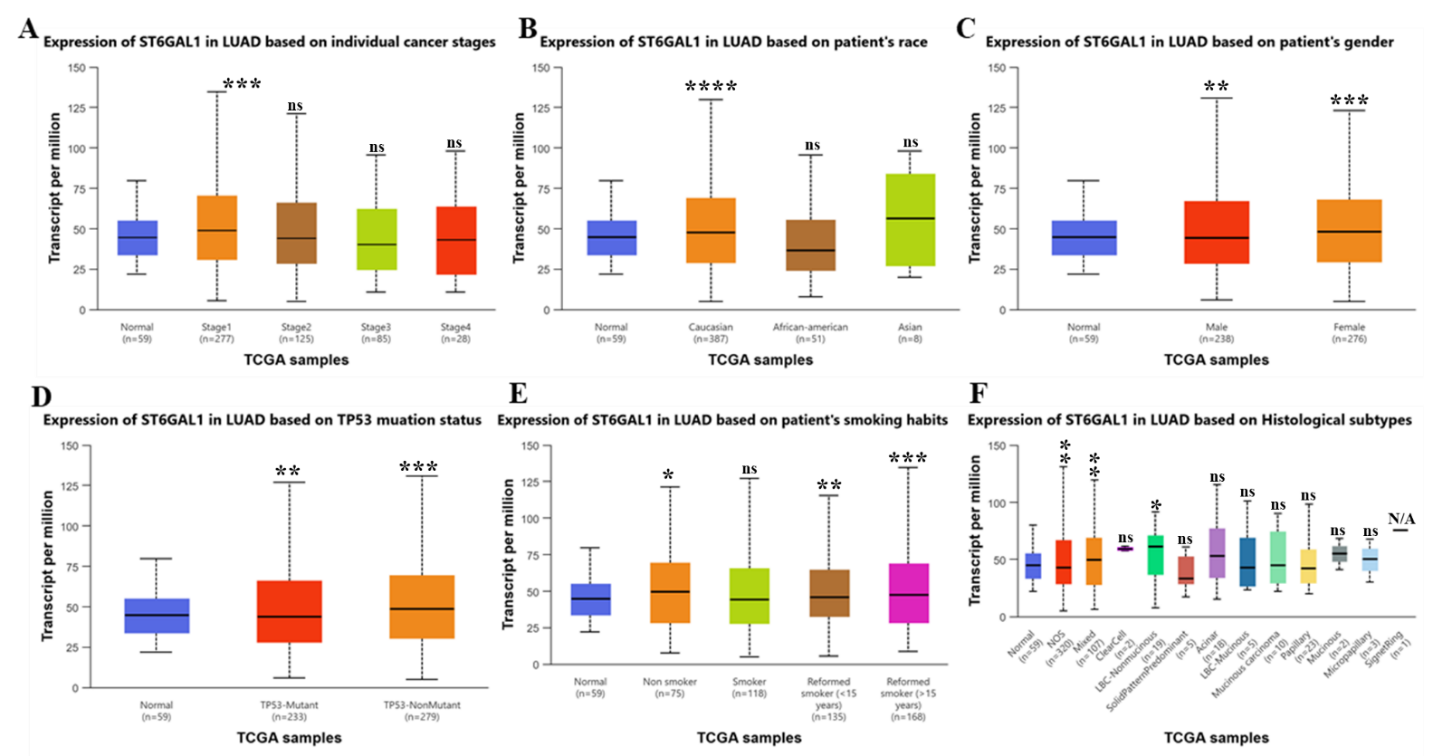
**Supplementary Figure S6. Clinical stratification of ST6GAL1 expression in lung adenocarcinoma.**** Boxplots showing **ST6GAL1 mRNA expression** (TPM) in LUAD and normal lung tissues derived from TCGA datasets and analyzed using UALCAN. (A) ST6GAL1 expression stratified by individual tumor stage (Stage I–IV). (B) ST6GAL1 expression according to patient race. (C) ST6GAL1 expression according to patient gender. (D) ST6GAL1 expression based on TP53 mutation status. (E) ST6GAL1 expression according to patients’ smoking history. (F) ST6GAL1 expression across different histological subtypes of LUAD. Statistical significance between groups is indicated as *P < 0.05, **P < 0.01, ***P < 0.001, ****P<0.0001; ns, not significant. N/A indicates comparisons that were not tested due to insufficient sample numbers.

**Supplementary Table S1: Complete gene list of the Glyco.marker signature.**

| **S.No** | **Glyco.marker** | **S.No** | **Glyco.marker** | **S.No** | **Glyco.marker** |
| --- | --- | --- | --- | --- | --- |
| **1** | **ALG1** | **39** | **GALNT1** | **77** | **SEC23B** |
| **2** | **ALG11** | **40** | **GALNT2** | **78** | **SEC24D** |
| **3** | **ALG12** | **41** | **GALNT4** | **81** | **SLC35G3** |
| **4** | **ALG3** | **42** | **GALNT6** | **82** | **ST3GAL1** |
| **5** | **ALG6** | **43** | **GALNT7** | **83** | **ST3GAL4** |
| **6** | **ALG8** | **44** | **GCNT1** | **84** | **ST6GAL1** |
| **7** | **ANXA3** | **45** | **GCNT3** | **85** | **SYS1** |
| **8** | **ATP11B** | **46** | **GOLGA3** | **86** | **TACSTD2** |
| **9** | **B3GALNT2** | **47** | **GPX4** | **87** | **TFAP2B** |
| **10** | **B3GALT6** | **48** | **HEY1** | **88** | **TM4SF1** |
| **11** | **B3GNT2** | **49** | **HOPX** | **89** | **TMED5** |
| **12** | **B4GALT1** | **50** | **HS3ST4** | **90** | **TMEM101** |
| **13** | **B4GALT5** | **51** | **HS6ST3** | **91** | **TMEM128** |
| **14** | **B4GALT7** | **52** | **ITIH2** | **92** | **TMEM139** |
| **15** | **B6GALNAC2** | **53** | **KRT13** | **93** | **TMEM207** |
| **16** | **C1GALT1** | **54** | **KRT19** | **94** | **TMEM9B** |
| **17** | **CD44** | **55** | **KRT4** | **95** | **TNF** |
| **18** | **CHST10** | **56** | **LGALS1** | **96** | **TNFSF10** |
| **19** | **CHST14** | **57** | **LGALS3** | **97** | **TNFSF9** |
| **20** | **CLDN16** | **58** | **LGALS7** | **98** | **UXS1** |
| **21** | **CLEC10A** | **59** | **LIN28B** | **99** | **VAMP3** |
| **22** | **COG4** | **60** | **MBL2** | **100** | **VAMP8** |
| **23** | **COG6** | **61** | **MGAT1** |  |  |
| **24** | **COLEC10** | **62** | **MGAT2** |  |  |
| **25** | **CTNNBIP1** | **63** | **MGAT4A** |  |  |
| **26** | **CXCL14** | **64** | **MGAT5** |  |  |
| **27** | **DPAGT1** | **65** | **MGAT5B** |  |  |
| **28** | **DPM1** | **66** | **MUC1** |  |  |
| **29** | **DPM2** | **67** | **NEU2** |  |  |
| **30** | **DUSP6** | **68** | **NOTCH1** |  |  |
| **31** | **EBAG9** | **69** | **NOTUM** |  |  |
| **32** | **EBPL** | **70** | **NT5E** |  |  |
| **33** | **FIBCD1** | **71** | **POMGNT1** |  |  |
| **34** | **FUT10** | **72** | **POMT1** |  |  |
| **35** | **FUT11** | **73** | **POU5F1B** |  |  |
| **36** | **FUT8** | **74** | **PRR11** |  |  |
| **37** | **GAL3ST3** | **75** | **RCN2** |  |  |
| **38** | **GALK1** | **76** | **S100A14** |  |  |
